# The cyclic nucleotide-gated channels CNGC2 and CNGC4 support systemic wound responses in *Arabidopsis thaliana*

**DOI:** 10.3389/fpls.2025.1545065

**Published:** 2025-08-22

**Authors:** Sarah Johns, Erin Wiegman, Arkadipta Bakshi, Simon Gilroy

**Affiliations:** Department of Botany, University of Wisconsin-Madison, Madison, WI, United States

**Keywords:** *Arabidopsis thaliana*, calcium, cyclic nucleotide gated channel 2 (CNGC2), cyclic nucleotide gated channel 4 (CNGC4), JAZ, systemic signaling, wounding

## Abstract

**Introduction:**

The local perception of a stimulus such as wounding can trigger plant-wide responses through the propagation of systemic signals including the vascular transport of diverse chemical messengers, the propagation of electrical changes, and even potentially hydraulic waves that rapidly spread throughout the plant body. These systemic signals trigger changes in second messengers such as Ca2+ that then play roles in triggering subsequent molecular responses. Although the glutamate receptor-like (GLR) channels GLR3.3 and GLR3.6 are known to be essential for the vascular propagation of wound-induced electrical and Ca2+ signals, additional channels and/or transporters are likely necessary to further spread responses across the plant. We hypothesized that members of the cyclic nucleotide-gated family of ion channels (CNGCs) might also be involved in the systemic component of this process.

**Methods:**

An analysis of the systemic induction of defense genes was made using qPCR and patterns of Ca2+ signaling were monitored in plants expressing the GFP-based Ca2+ sensor GCaMP. Wild-type responses were compared to those seen from a library of CNGC mutants.

**Results:**

Of all the CNGC family members tested, only mutants in CNGC2 and CNGC4 showed disruption in the patterns of both leaf-to-leaf and root-to-leaf wound-triggered systemic induction of defense gene expression. The mutants in these channels showed wild-type-like propagation of Ca2+ increases from the wound site but exhibited a limited spread of the Ca2+ wave from the vasculature to other tissues of distal leaves.

**Discussion:**

CNGC2 and CNGC4 likely play roles in spreading the Ca2+ signal through systemic leaves to help further propagate and amplify the plant-wide wound response. Although CNGC19 has previously been shown to be involved in Ca2+ signaling at the wound site, knockouts in this gene did not disrupt the long-distance element of the wound response. These findings suggest that the molecular machinery required to trigger the local reaction to damage is likely, at least in part, distinct from the activities that support the systemic spread of the response throughout the plant.

## 1 Introduction

Rapid, long-distance signals are used by plants to integrate responses throughout the plant body—for example, local perception of heat, cold, herbivory, wounding, and pathogens all elicit plant-wide signaling which results in changes ranging from the modification of patterns of gene expression and shifts in cellular chemistry to alterations in growth and development (Fromm and Lautner, 2007; Vodeneev et al., 2015; Shabala et al., 2016). Electrical and hydraulic signals, waves of Ca^2+^ and reactive oxygen species (ROS), and the mass flow of chemical signals in the vasculature have all emerged as elements of the systems that propagate these rapid, long-distance signals (reviewed in Notaguchi and Okamoto, 2015; Gilroy et al., 2016; Farmer et al., 2020; Johns et al., 2021; Ravi et al., 2023). Despite these advances in our understanding of what factors are carrying information through the plant, precisely how they then propagate and trigger subsequent responses at a systemic level remains to be fully characterized.

Glutamate receptor-like (GLR) channels 3.1, 3.2, 3.3, and 3.6 are all implicated in the systemic signaling network to wounding as knockout mutants of all four genes exhibit altered signaling triggered by herbivory or mechanical damage—for example, the *glr3.3/3.6* double knockout mutant severely disrupts the wound-induced systemic propagation of both electrical and Ca^2+^ signaling (e.g., Mousavi et al., 2013; Nguyen et al., 2018; Toyota et al., 2018; Wu et al., 2024). GLR3.3 and GLR3.6 have been confirmed to be present in the sieve tube elements and companion cells and in the xylem contact cells, respectively (Nguyen et al., 2018; Wu et al., 2024). These patterns of expression are consistent with their acting in a system known to facilitate the rapid spread of the systemic wound signal via the vasculature (e.g., Bellandi et al., 2022; Gao et al., 2023; Grenzi et al., 2023). However, the observation that the vascular-expressed, mechanosensitive ion channel mechanosensitive channel of small conductance-like 10 (MSL10) acts alongside GLR3.3 and 3.6 in the systemic propagation of wound response (Moe-Lange et al., 2021) highlights the likely roles for additional ion transporters and channels in this system. In particular, the molecular machinery involved in the movement of the systemic responses to tissues beyond the vasculature remains largely undefined.

In *Arabidopsis*, the cyclic nucleotide-gated channels (CNGCs) are a 20-member family of cation channels that show a range of ion permeabilities, including Ca^2+^ (**Supplementary Figure S1**; reviewed in Mäser et al., 2001; Jammes et al., 2011). They are thought to function in multiple aspects of plant development and to mediate responses to both biotic and abiotic stresses (Mäser et al., 2001; Steinhorst and Kudla, 2013)—for example, mutants in *CNGC2* and *CNGC4* exhibit phenotypes ranging from a reduced hypersensitive response and constitutively high pathogen defense to reduced vegetative growth, with a *CNGC2* knockout exhibiting disrupted systemic signaling to high light stress (e.g., Yu et al., 1998; Jurkowski et al., 2004; Wang et al., 2017, 2019; Tian et al., 2019; Fichman et al., 2021; Zhao et al., 2021). CNGC19 has also been shown to contribute to plant responses to mechanical damage and herbivory and participate in immune response and programmed cell death (Meena et al., 2019; Yu et al., 2019; Jogawat et al., 2020). Similarly, CNGC17 is known to form a complex with core components of immune defense signaling (Ladwig et al., 2015).

Although there is much evidence for CNGC involvement in plant stress and defense response networks, little is known about any relationship to the systemic aspects of signaling. We therefore used mutants in members of the CNGC family to explore their potential role(s) in long-distance wound signaling in *Arabidopsis*. Of the 17 CNGCs tested, only mutants in *CNGC2* and *CNGC4* disrupted the systemic molecular responses to wounding. An analysis of the dynamics of wound-induced Ca^2+^ increases in the *CNGC2* and *CNGC4* knockout backgrounds suggests that these channels are involved in supporting the spread of a Ca^2+^ signal radiating from the vasculature once the systemic wound signal has arrived in distal leaves.

## 2 Methods

### 2.1 Plant material

The CNGC family consists of 20 members (**Supplementary Figure S1**), and mutants in 17 were obtained either from the Arabidopsis Biological Resource Center (ABRC, Ohio State University) or as generous gifts from collaborators (**Supplementary Table S1**). Lines were validated by PCR using the primers listed in **Supplementary Table S2**. We were unable to obtain homozygous lines in *CNGC*7 and CNGC*18*. However, as these are pollen-expressed channels (as are *CNGC8* and *CNGC16*), they might be expected to have minimal influence on wound signaling within the roots and leaves which are the focus of our investigation. Unfortunately, we were also unable to validate a homozygous knockout in *CNGC15*, which is expressed in vegetative tissue. Although we have focused on single mutants in our analysis, a *cngc10/13* double mutant was a kind gift from Dr. Keiko Yoshioka (University of Toronto, Canada) and was included in our analysis. In general, these mutant lines grew similarly to wild type with no obvious disruption of growth and development, except for knockouts in *CNGC2* and *CNGC4* which exhibited the slower growth patterns already reported in the literature (Ma et al., 2023).

### 2.2 Systemic and root-to-shoot wound assays

For leaf-to-leaf systemic signaling, plants were grown on soil for 6 to 7 weeks under short day conditions (8 h of light, 16 h of darkness) at 22°C and 70% humidity, except for the mutants in *CNGC2* and *CNGC4* which grow slightly more slowly and so required 8 to 9 weeks of growth to reach a usable size. We applied the leaf numbering scheme outlined in **Supplementary Figure S2**, where the oldest leaf was designated as leaf 1 and higher numbers reflect progressively younger leaves. Leaf (*n*) was wounded by crushing across the width of the lamina with serrated tweezers and leaves (*n* + 4) and (*n* + 5) were then collected at 0 and 40 min and immediately frozen in liquid nitrogen. Four biological replicates (individual plants) were analyzed per genotype and per timepoint for each independent experiment. For mutants in *CNGC2* and *CNGC4*, the leaves were pooled from three plants per biological replicate to yield sufficient tissue for RNA extraction due to their small size.

For root-to-shoot signaling assays, the plants were grown vertically for 2 weeks on Petri plates containing ½ strength Linsmaier and Skoog media (½ LS; PhytoTech Labs) with 0.3% (w/v) sucrose and 1% (w/v) Phytagel (Sigma-Aldrich) under 12 h of light at 22°C. The main root was then crushed ~1 cm from the shoot’s base with serrated tweezers. The entire rosette was collected at 0 and 40 min timepoints as for the leaf wounding experiments described above.

### 2.3 Quantitative PCR

Total RNA was extracted using the TRIzol Reagent (Invitrogen) according to the manufacturer’s instructions. Briefly, sample tissue was ground for 30 s at 1,500 rpm using the MiniG Automated Tissue Homogenizer Model 1600 (SPEX SamplePrep), shaken with TRIzol for 5 min, vortexed with a mixture of 24:1 (v/v) chloroform/isoamyl alcohol, and centrifuged at 14,000 rpm. Nucleic acid was precipitated using isopropanol and washed with 75% (v/v) ethanol, and the pellet was resuspended in sterile water. Contaminating DNA was removed using TURBO DNAse (Invitrogen) according to the manufacturer’s instructions. Quantitative PCR was performed using the Luna Universal One-Step RT-qPCR Kit (New England Biolabs) according to the manufacturer’s instructions with the primers described in **Supplementary Table S3**. Gene expression levels were normalized to the expression levels of *UBIQUITIN10* using the ΔΔCT method (Choi and Roberts, 2007) and normalized to fold-change versus the 0 min timepoint in wild-type plants for root-to-shoot assays and the 0 min timepoint of leaf 14 (*n* + 4) in the systemic leaf wounding assays. Either one-way ANOVA with *post-hoc* Tukey HSD test or Student’s *t*-test was used in the statistical analyses, both with a critical value of 0.05.

### 2.4 Generation of GCaMP3-expressing lines

Multiple independent reporter lines ubiquitously expressing the fluorescent Ca^2+^ bioreporter GCaMP3 (driven by the 35S promoter) in the *cngc19–1* and *cngc20* mutant backgrounds were generated using *Agrobacterium* floral dip, as described in Toyota et al. (2018). After at least three generations of selfing, bright, non-segregating lines that showed no overt effects on growth and development were selected for further analysis (the two transformed lines used in this work are denoted *cngc19–1 g-1* and *cngc20 g-6*). The transformation of *cngc2–1* and *cngc4–1* was attempted but failed multiple times. Therefore, *cngc2–1* and *cngc4-1* (and the *cngc19–1* and *cngc20* mutants) were also crossed with the Col-0 GCaMP3 line from Toyota et al. (2018). Although GCaMP expressing *cngc19–1* and *cngc20* lines were readily obtained using this approach (denoted *cngc19–1 C* and *cngc20 C* in the results), the success of crossing and subsequent seed set for *cngc2–1* and *cngc4–1* was very low. However, homozygous *cngc2–1* and *cngc4–1* reporter-expressing lines were eventually obtained. All crossed reporter lines were used in the F3 or higher generation.

### 2.5 Fluorescence microscopy

Plants expressing GCaMP3 were grown on ½ LS with 0.3% (w/v) sucrose and 1% (w/v) Phytagel for 2 weeks. Whole plants were visualized using a Zeiss AxioZoom microscope with ×1/0.25 lens set at ×7 zoom with 470/40-nm excitation, 500-nm dichroic mirror, and 535/50-nm emission. The root or leaf was wounded by crushing with tweezers, and images were recorded every 2 s starting 1 min pre-wounding and continuing until the changes in fluorescence had dissipated. For the higher-magnification microscopy needed to monitor the spread of the Ca^2+^ wavefront from the leaf vasculature the ×1 lens was set at either ×16 or ×20 zoom. Fluorescence values were obtained using ImageJ (Schneider et al., 2012). Change in fluorescence was calculated after subtraction of the averaged background measured from the growth medium and was normalized by calculating F/F_0_, where F is the fluorescence at each timepoint and F_0_ is the average fluorescence for the 1 min before the wounding event. The time when the change in fluorescence reached two standard deviations above the background, the peak change in fluorescence and the timing of the end of the signal were all recorded. Quantitative fluorescence analysis was performed directly from the Zeiss.czi image files imported into ImageJ. However, for some lines, the fluorescence signal was dim (most notably *cngc2–1* and *cngc4–1* GCaMP3-expressing lines). Therefore, during post-quantitative analysis, for all display panels in the figures, the maximum upper display limit of the raw images from the AxioZoom was uniformly and linearly decreased by half within ImageJ for ease of viewing. When the display limit is similarly adjusted in a **Supplementary Video**, it is noted in the figure legend. The raw.czi imaging files for these figures and videos are all available at: https://doi.org/10.6084/m9.figshare.28017179.v1.

Calcium wave extent was determined using ImageJ. Individual images from each time-lapse series were aligned using the StackReg plugin with Rigid Body transformation to account for any minor leaf movement during the time course measurement. Wave width was calculated by drawing a line perpendicular to either the first-order (midvein), second-order, and third-order veins of the leaf and the Plot Profile tool used to record the GCaMP3 fluorescence intensity values along these transects. Wave extent was defined as the distance where the fluorescence intensity had continuously risen to at least two standard deviations above the pre-wounding resting signal measured from the surface of the vein to the edge of the wave. Two standard deviations represent the 95% confidence interval for the pre-wounding signals. Datapoints were measured every five frames (i.e., at 10-s intervals) of the time course, and the maximum distance of the wavefront was used for statistical analysis to compare the Ca^2+^ wavefront extent between wild-type and mutant plants.

## 3 Results

### 3.1 Screening the CNGCs for roles in long-distance signaling

As noted above, the CNGCs represent potential candidates for channels facilitating the systemic spread of the wound response, especially as CNGC19 has been shown to be involved in generating Ca^2+^ signals in wounded leaf tissue (Meena et al., 2019). As the CNGCs form a 20-member gene family in *Arabidopsis* (summarized in **Supplementary Figure S1**), we initiated a mutant screen to ask which were potentially playing a role in systemic wound response. As CNGCs 7, 8, 16, and 18 form two pollen-expressed groups of channels (**Supplementary Figure S1**), we reasoned that they are unlikely to be central players in wound responses in the vegetative tissues of the plant and so we assayed one representative mutant from each group (CNGC8 from CNGC group II and CNGC16 from group III). Of the remaining 16 CNGCs, we were able to validate mutants in 15 (all except *CNGC15*) and used this library of lines to characterize their effects on systemic transcriptional response to wounding of either the leaf or the root.

**Figure 1:**
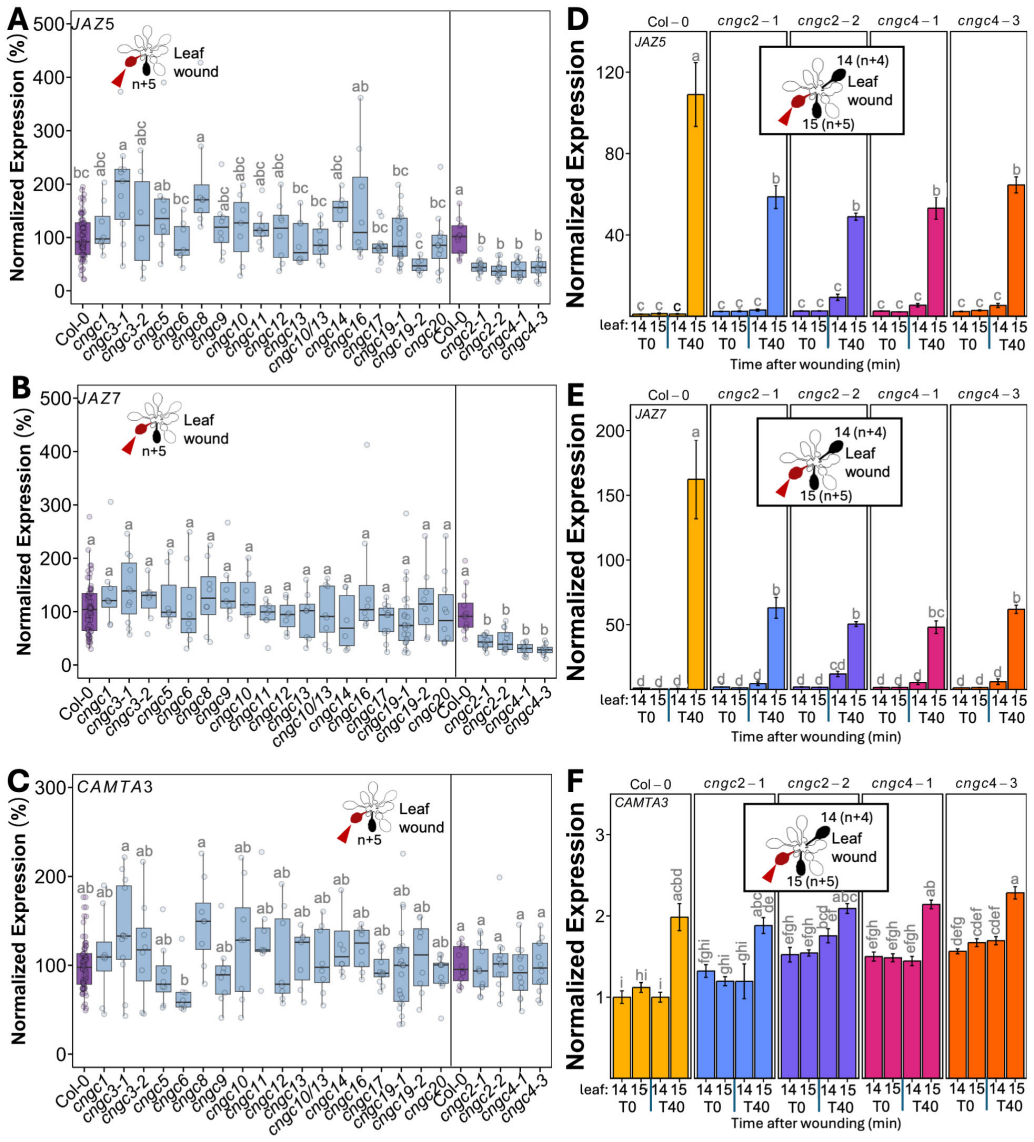
Mutants in *CNGC2* and *CNGC4* disrupt the systemic induction of leaf wound-triggered gene expression. Expression of *JAZ5* **(A)**, *JAZ7* **(B)**, and *CAMTA3* **(C)** in leaf 15 at 40 min post-wounding in *cngc* mutants presented as fold-induction relative to the Col-0 level averaged for two to three independent experiments (*n* = 9–12), i.e., 100% would represent a response identical to wild type: 100-fold wound-induced increase in *JAZ5*, 150-fold increase in *JAZ7*, and twofold increase in *CAMTA3*. Data and statistical analysis for *cngc2-1*, *cngc2-2*, *cngc4-1*, and *cngc4–3* with Col-0 for their experimental replicates shown separately as an extra 2 weeks of growth was necessary for sufficient leaf size for analysis in these mutants. **(D–F)** Representative experiment from **(A-C)** showing the systemic expression of **(D)** *JAZ5*, **(E)** *JAZ7*, and **(F)** *CAMTA3* following the wounding of leaf 10 in *cngc2-1*, *cngc2-2*, *cngc4-1*, and *cngc4–3* monitored at 0 (T0) and 40 (T40) min post-wounding. The expression is normalized to Col-0 leaf 14 at 0 min post-wounding. The data is mean ± SEM of three to four biological replicates with three technical replicates each. Bars with different letters are significantly different from each other (*p* < 0.05) based on ANOVA with multiple comparisons via Tukey.

The spread of systemic wound responses in the *Arabidopsis* rosette has been shown to be bidirectional, with younger leaves signaling to older leaves and vice versa (Toyota et al., 2018). Therefore, to characterize CNGC effects on leaf-to-leaf systemic signaling, we wounded an older leaf and assayed responses in younger leaves. *Arabidopsis* rosette development can be described by numbering the leaves as they form from the oldest to the youngest (**Supplementary Figure S2**). Using this classification scheme, previous research has shown that upon wounding leaf (*n*), the strongest systemic changes in a suite of responses that include increases in Ca^2+^ level, changes in wound-linked surface potential, and induction of defense responses are observed in leaf *n* + 5 (a leaf on the same side of the rosette as the wounded leaf). Despite being developmentally very close in age to leaf *n* + 5, little to no response is seen in leaf *n* + 4 on the opposite side of the rosette (Mousavi et al., 2013; Nguyen et al., 2018; Toyota et al., 2018). We capitalized on this *n* + 5/*n* + 4 patterning by wounding leaf 10 and cataloging the patterns of defense gene induction in leaves 14 and 15. We used qPCR to monitor the induction of the classic wound and jasmonic acid-responsive genes *JASMONATE-ZIM-DOMAIN PROTEIN 5* (*JAZ5*) and *JAZ7* (Chung et al., 2008) along with *CALMODULIN-BINDING TRANSCRIPTIONAL ACTIVATOR 3* (*CAMTA3*). CAMTA3 has been linked to stress response pathways such as immune signaling, cold shock, and touch (e.g., Doherty et al., 2009; Kim et al., 2017; Darwish et al., 2022) but does not lie in the canonical jasmonic acid-linked systemic wound signaling pathway. Thus, this gene provided us with a control marker for the selectivity of activation of systemic stress responses by our wounding treatments. Critically, we first confirmed that our system indeed followed the *n* + 4/*n* + 5 patterning of response. **Figure 1** shows that at 40 min after wounding of leaf 10 of wild-type plants, a significant (*p* < 0.05) >100-fold induction in *JAZ5* (**Figures 1A, D**) and *JAZ7* (**Figures 1B, E**) was detectable in leaf 15 (*n* + 5) but not in leaf 14 (*n* + 4). In addition, this treatment failed to elicit a statistically significant change in *CAMTA3* levels, confirming the applicability of this gene as a control (**Figures 1C, F**).

**Figure 2:**
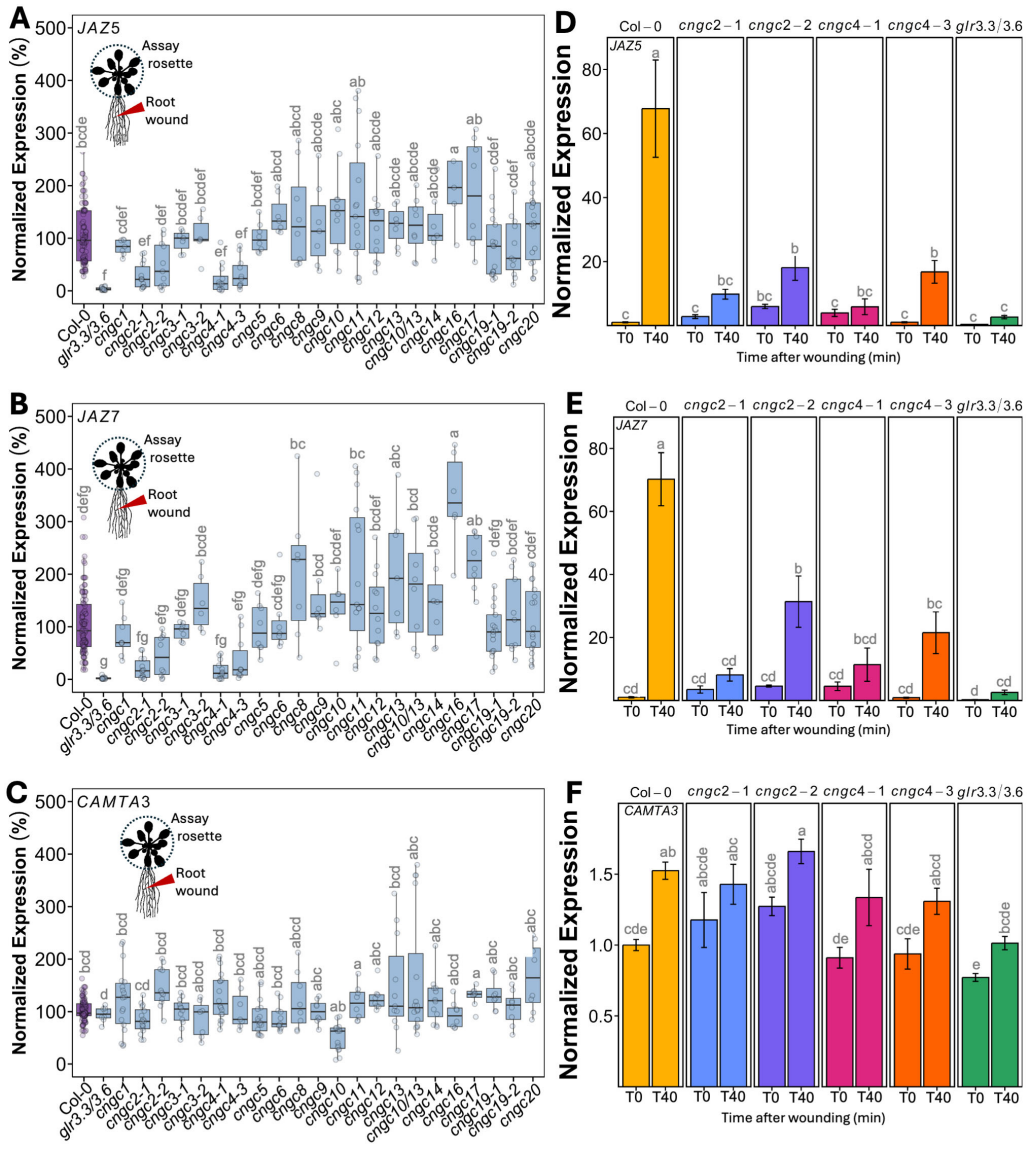
Mutants in *CNGC2* and *CNGC4* disrupt the systemic induction of root wound-triggered gene expression. **(A–C)** Expression of *JAZ5* **(A)**, *JAZ7* **(B)**, and *CAMTA3* **(C)** in shoot tissue (whole rosette) at 40 min post-root wounding in *cngc* mutants presented as fold-induction relative to the Col-0 level averaged for two to three independent experiments (*n* = 10–12), i.e., 100% would represent a response identical to wild type: 70-fold wound-induced increase in *JAZ5*, 70-fold increase in *JAZ7*, and 1.5-fold increase in *CAMTA3*. **(D–F)** Representative experiment showing the systemic expression of **(D)** *JAZ5*, **(E)** *JAZ7*, and **(F)** *CAMTA3* following the wounding of the root of *cngc2-1*, *cngc2-2*, *cngc4-1*, and *cngc4–3* monitored at 0 (T0) and 40 (T40) min post-wounding. The expression is normalized to Col-0–0 min post-wounding. The data is mean ± SEM of three to four biological replicates with three technical replicates each. Bars with different letters are significantly different from each other (*p* < 0.05) based on ANOVA with multiple comparisons via Tukey.

We were also interested in how root-to-shoot systemic wound signaling might similarly be operating as there is relatively little literature describing this phenomenon. We adopted a similar qPCR-based analysis, wounding the primary root and then following patterns of marker gene expression in the shoot tissues (assaying the entire rosette). **Figure 2** shows that in wild-type plants, such root wounding triggered a ~70-fold induction of *JAZ5* (**Figure 2D**) and *JAZ7* (**Figure 2E**) expression in the rosette. Notably, although CAMTA3 showed a wound-induced systemic induction, it was only up to ~1.5-fold (**Figure 2F**).

Having validated our qPCR-based assay, we systematically applied wounding to a library of *CNGC* mutants with the results of leaf-to-leaf responses shown in **Figure 1** and root-to-shoot in **Figure 2**. It is important to note that wounding experiments have a degree of variability as it is challenging to reproduce identical mechanical wounds when experimental replicates are performed on different days. This means that although the overall patterns of response (e.g., upregulation vs. downregulation of marker genes) are generally highly reproducible across independent experiments, absolute levels of gene induction can vary from experiment to experiment. Therefore, we provide results showing these systemic patterns of wound-induced marker gene response in two ways. First, we show a summary of the magnitude of marker gene induction of the responding leaf (leaf 15) at 40 min post-wounding of leaf 10, with the response averaged across all independent experimental replicates (**Figures 1A–C**). To make this average, we normalized the responses in each mutant to the wound-induced expression level of each marker gene seen in the wild type in each individual experiment (i.e., mutant expression in leaf 15 at 40 min post-wounding relative to wild type). We then compared these normalized responses across all independent experiments. In this manner, a mutant showing a response of 100% in this summary analysis (**Figures 1A–C**) would have an average level of wound-induced transcriptional response across all experimental replicates identical to wild type. Mutants with values more or less than 100% would then be exhibiting a greater or lesser level of gene induction than the wild type across all experiments. This approach allows a ready comparison of the extent of the average wound-induced fold-induction of defense gene induction in leaf 15 of the mutants to wild type while accounting for replicate-to-replicate variability in absolute levels of expression. **Figures 2A–C** show a similar normalized summary analysis for root-to-shoot wounding experiments. However, for both the leaf and root wounding experiments, a second representation of these same experiments is needed to show the time component of induction (i.e., comparing expression at 0 min vs. 40 min post-wounding) and the spatial patterning (comparing leaves 14 to 15). Therefore, a representative result from a single experiment is also shown (**Figures 1D–F**, **2D–F**). Here the expression has been normalized to the wild-type control for that particular experiment. Although **Figures 1D–F** and **2D–F** only show these representative experimental data for mutants in *CNGC2* and *CNGC4*, the equivalent representative experimental data for all of the other CNGCs tested is available in **Supplementary Figures S3–S6**. The raw qPCR data for all of these experiments is also available on Figshare (https://doi.org/10.6084/m9.figshare.28017227.v1).

To identify mutants with a significant difference in systemic transcriptional response, we applied a threshold of requiring a significant twofold change in at least two of the marker genes *JAZ5*, *JAZ7*, and *CAMTA3* across all experimental replicates. Using this criterion, the systemic transcriptional responses of *CNGCs 1*, *3*, *5*, *6*, *8*, *9*, *10*, *11*, *12*, *13*, *14*, *16*, *17*, *19*, and *20* and a double mutant in *CNGC*s *10* and *13* were not significantly different from the wild type following either leaf or root wounding.

### 3.2 Knockouts of *CNGC2* and *CNGC4* alter systemic signaling

However, in our screening of CNGCs for effects on the systemic induction of wound response to local damage, knockouts in *CNGC2* and *CNGC4* stood out as the only plants to show a significant alteration in systemic transcriptional responses. Thus, *cngc2-1*, *cngc2-2*, *cngc4-1*, and *cngc4–3* had a >50% reduction in leaf wound-triggered induction in both *JAZ5* and *JAZ7* transcripts in leaf 15 at 40 min post-wounding of leaf 10, with no significant change in *CAMTA3* expression (**Figures 1A–C**). These mutants were also impaired in root-to-shoot wound responses, with, again, a significant >50% reduction in wound-induced *JAZ5* and *JAZ7* expression in the rosette upon wounding the root and with patterns of *CAMTA3* expression resembling the minimal changes seen in the wild type (**Figures 2A–C**). For comparison, the double knockout mutant, *glr3.3/3.6* was included as a positive control for a mutant background known to robustly inhibit wound-induced systemic transcriptional outputs. No statistically significant differences were observed in the magnitude of reduced *JAZ5* and *JAZ7* responses between the *glr3.3/3.6* mutant and the *cngc2–1* or *cngc4–1* alleles analyzed (**Figures 2A–F**), implying important roles for CNGC2 and CNGC4 in these responses. However, there were small but statistically significant increases in *JAZ5* and *JAZ7* induction in the *cngc2–2* and *cngc4–3* backgrounds compared to *glr3.3/3.6* (**Figures 2D, E**).

### 3.3 *cngc19* and *cngc20* show a similar systemic Ca^2+^ response to the wild type

Previous research has shown that a propagating Ca^2+^ increase is triggered by local leaf wounding and is part of the signaling system leading to systemic molecular responses such as *JAZ* gene induction (Nguyen et al., 2018; Toyota et al., 2018). Thus, CNGC mutant effects on the dynamics of this systemic Ca^2+^ wave might explain their disruption of systemic defense gene induction. Furthermore, this wound-induced systemic change in Ca^2+^ is known to obey the *n* + 5/*n* + 4 patterning in the wild-type rosette (Nguyen et al., 2018; Toyota et al., 2018), providing a clear wild-type spatial Ca^2+^ signature for comparison with those seen in the mutants. In contrast, the effects of root wounding on systemic Ca^2+^ signals are not well characterized. We therefore used plants ubiquitously expressing the GFP-based Ca^2+^-reporter GCaMP3 to characterize the Ca^2+^ signals triggered by wounding in both the wild type and *CNGC* knockouts.

**Figure 3:**
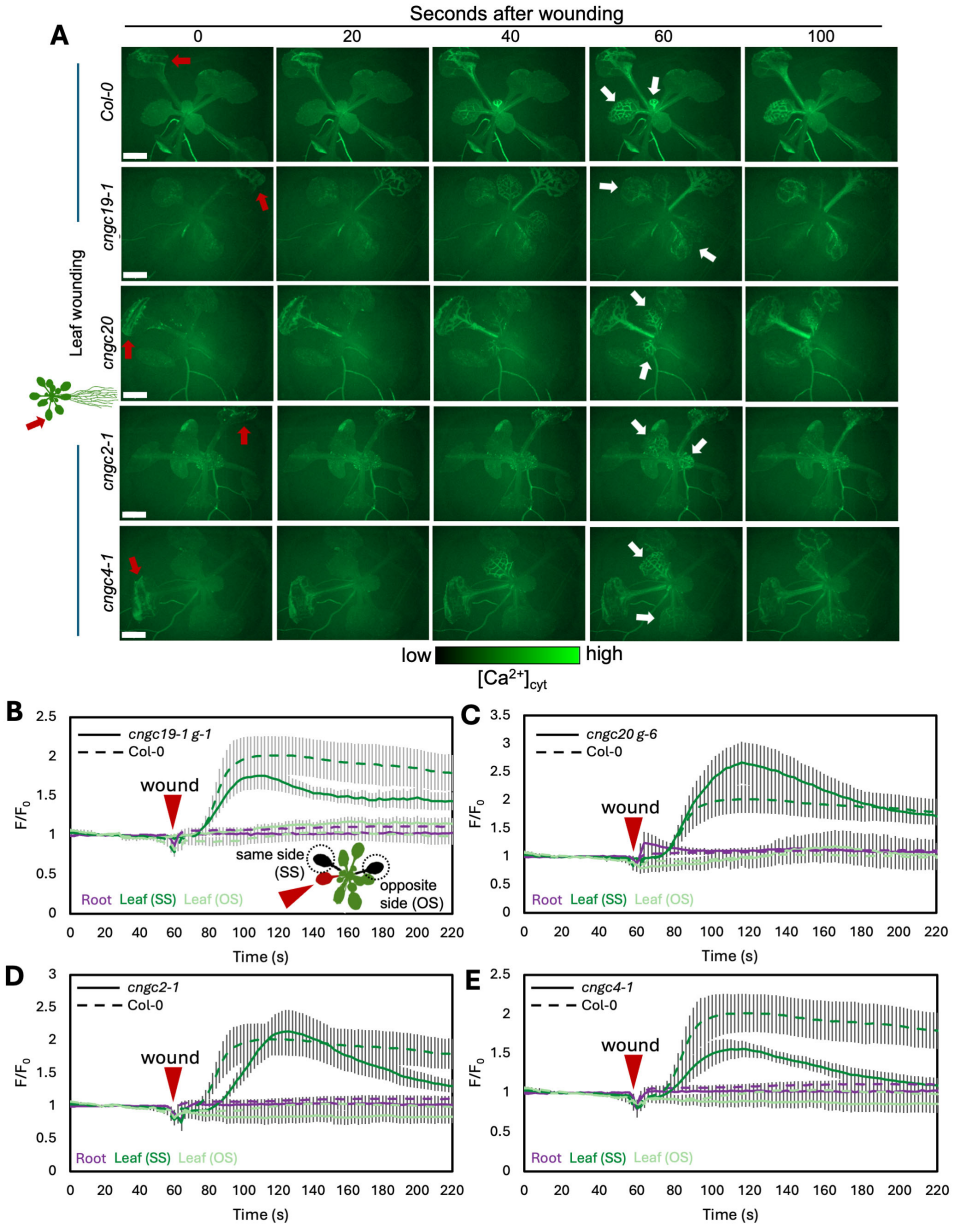
Ca^2+^ wave following leaf wounding in Col-0, *cngc19-1*, *cngc20*, *cngc2-1*, and *cngc4-1*. **(A)** After wounding of the leaf (red arrow), a wave of increased Ca^2+^ travels to the leaves on the same side (SS) of the rosette as the initial wound, but not to the leaves on the opposite side (OS), or into the root system. White arrows indicate the examples of responding leaves. Ca^2+^ levels visualized by the GCaMP3 fluorescent biosensor ubiquitously expressed in Col-0, *cngc19-1*, *cngc20*, *cngc2-1*, and *cngc4-1*. Representative of *n* = 8–11 (see **Supplementary Videos S1, S3, S5, S7**, **S8**). Scale bars, 2 mm. **(B–E)** Quantification of the normalized fluorescence (F/F_0_) of the main root (purple) and leaves: systemic leaf on the same side of the rosette as the wound (SS; dark green) and systemic leaf on the opposite side (OS; light green) for *cngc19-1* **(B)**, *cngc20* **(C)**, *cngc2-1* **(D)**, and *cngc4-1* **(E)**, with solid lines indicating the response of each of the mutants and dashed lines representing the same Col-0 control data in each panel for comparison. F, fluorescence intensity; F_0_, average fluorescence before wound stimulus where an increase in F/F_0_ represents an increase in Ca^2+^ levels. The results are mean ± SEM, *n* = 8–10. Reporter lines *cngc19–1 g-1* and *cngc20 g-6* were produced by transformation with the GCaMP3 reporter. Equivalent data is shown in **Supplementary Figure S7** showing a similar response in additional reporter lines made by crossing to the GCaMP3 expressing wild type.

**Figure 4:**
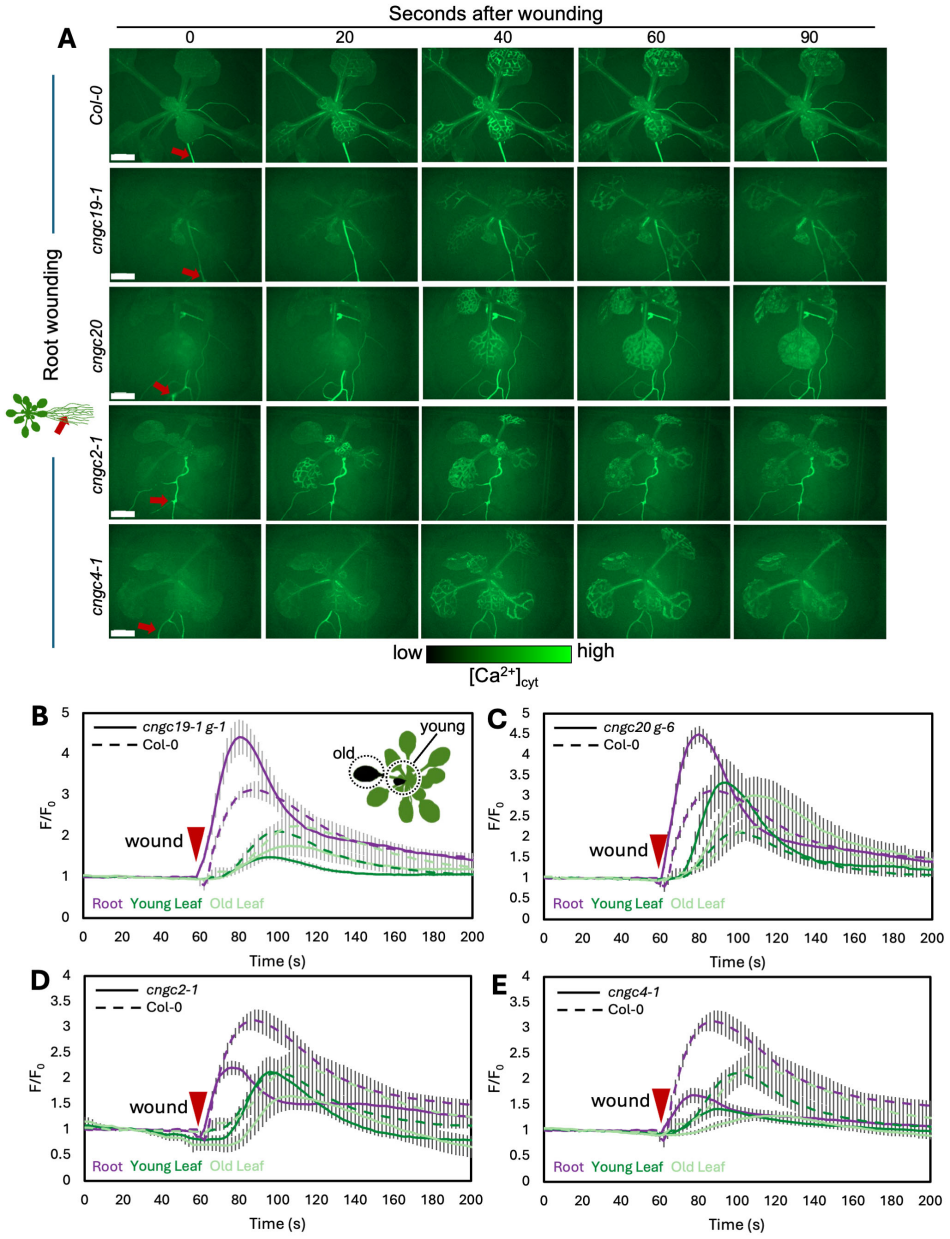
Ca^2+^ wave following root wounding in Col-0, *cngc19-1*, *cngc20*, *cngc2-1*, and *cngc4-1*. **(A)** After wounding of the main root (red arrow), a wave of increased Ca^2+^ levels travels to all leaves of the rosette as visualized by the GCaMP3 fluorescent biosensor ubiquitously expressed in Col-0, *cngc19–1 g-1*, *cngc20 g-6*, *cngc2-1*, and *cngc4-1*. Representative of *n* = 8–13 (see **Supplementary Videos S2, S4, S6, S9**, and **S10**). **(B–E)** Quantification of the normalized fluorescence (F/F_0_) of the main root (purple), young leaf (dark green), and older leaf (light green) for *cngc19–1 g-1* **(B)**, *cngc20 g-6* **(C)**, *cngc2-1* **(D)**, and *cngc4-1* **(E)**, with solid lines indicating the response of each of the mutants and dashed lines representing the same Col-0 control data in each panel for comparison. F, fluorescence intensity; F_0_, average fluorescence before wound stimulus where an increase in F/F_0_ represents an increase in Ca^2+^ levels. The results are mean ± SEM, *n* = 8–13. Scale bars, 2 mm.

We first confirmed that wounding a single leaf in the rosette of a wild-type plant led to the expected systemic spread of a wave of increased Ca^2+^ favoring responses in leaves on the same side of the rosette to those on the opposite side, as predicted from the *n* + 4/*n* + 5 patterning (**Figures 3A, B**; **Supplementary Video S1**). In addition, we found that when the wild-type root is wounded, a wave of Ca^2+^ response propagates shootward along the root and then spreads through the entire rosette, suggesting a widespread aerial systemic response (**Figures 4A, B**; **Supplementary Video S2**) consistent with the induction of the rosette-level molecular responses we observed to be induced by root wounding as discussed above (**Figures 2A, B, D, E**).

**Figure 5:**
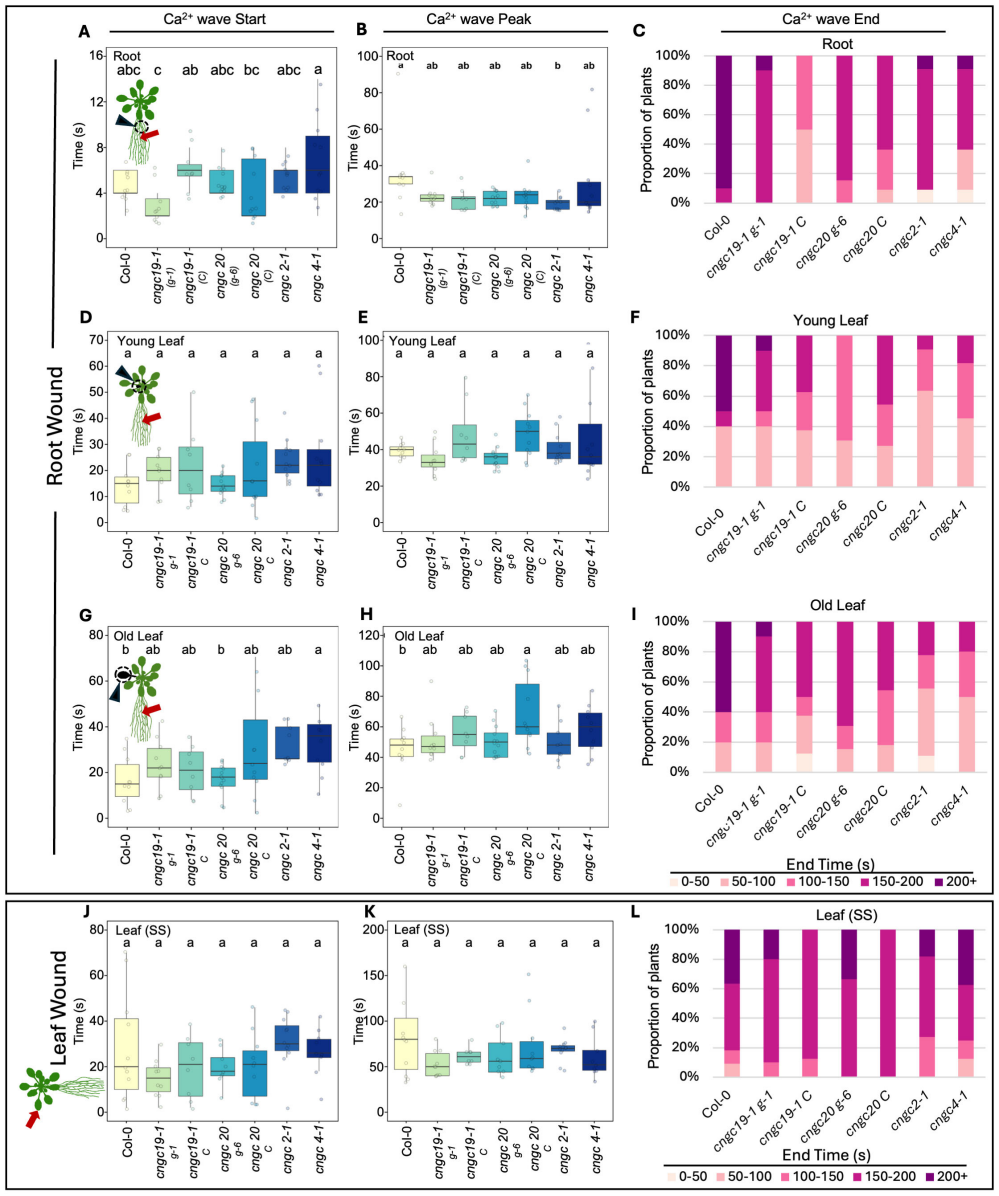
Timing of the start, peak, and end of the Ca^2+^ wave following root or leaf wounding in Col-0, *cngc19-1*, *cngc20*, *cngc2-1*, and *cngc4-1*. After wounding of the main root, the length of time post-wounding (s) it takes for the Ca^2+^ wave to start, peak, and then return to background (end) was monitored in Col-0 (wild type), *cngc19–1 g-1*, *cngc19–1 C*, *cngc20 g-6*, *cngc20 C cngc2-1*, and *cngc4-1.* In response to root wounding, analysis is shown for Ca^2+^ changes in root base **(A–C)**, young leaves **(D–F)**, and older leaves **(G–I)**. For leaf wounding **(J–L)**, the Ca^2+^ wave parameters are shown for the responding leaf (i.e., leaf on the same side of the rosette as the wounded leaf). Bars with different letters are significantly different from each other (*p* < 0.05) based on ANOVA with multiple comparisons via Tukey. End times were variable and are listed as percentages of the replicates returning to ≤baseline F/F_0_ within each 50s- interval up to 200 s (200+ reflects replicates that failed to return to background within 200 s). Data compiled from the images in **Figures 3** and **4** and additional Ca^2+^ imaging data deposited at Figshare (https://doi.org/10.6084/m9.figshare.28017179.v1). Ca^2+^ measurements were made using the GCaMP3 fluorescent biosensor. *cngc19–1 g-1* and *cngc20 g-6* were lines independently transformed with the reporter; all other lines were generated by crossing to the Col-0 GCaMP3 reporter line.

CNGC19 is known to play a key role in the Ca^2+^ changes and anti-herbivore defense linked to local wounding (Meena et al., 2019). CNGC19 is also characterized as forming heteromeric channels with its fellow CNGC Group IVa member, CNGC20 (Yu et al., 2019), although only knockouts in CNGC19 compromise anti-herbivory responses (Meena et al., 2019). However, neither knockouts in CNGC19 nor in CNGC20 showed a disruption of wound-induced transcriptional responses in our screen of leaf-to-leaf (**Figures 1A–C**, **Supplementary Figure S3**) or root-to-shoot (**Figures 2A–C**, **Supplementary Figure S4**) analyses. This absence of a discernible impact of CNGC19 knockout on systemic transcriptional responses to wounding prompted us to investigate whether systemic Ca²^+^ signaling might exhibit alterations in these mutants that were not reflected in the molecular markers of wound responses. We therefore assayed multiple reporter lines of *cngc19–1* and *cngc20* expressing the GFP-based Ca^2+^ reporter GCaMP3 in both leaf- and root-wounding experiments. We observed that systemic Ca^2+^ response was very similar to the wild type in both knockout backgrounds (**Figures 3A–C**, **4A–C**; **Supplementary Videos S3–S6**)—for example, wild type and both *cngc19–1* and *cngc20* showed the onset of Ca^2+^ increase in the systemic leaves starting ~20 s after wounding, which peaks 40 s later (**Figure 5**; **Supplementary Figures S7**, **S8**), and where the initial spread is most evident within the vasculature (**Figures 3A**, **4A**). Taken together, these observations of wild-type-like transcriptional response and largely wild-type-like Ca^2+^ wave propagation in response to root and shoot wounding suggest that even though CNGC19 is well characterized as being important for the generation of Ca^2+^ signals at the site of herbivore attack (Meena et al., 2019), it may not be as essential for triggering Ca^2+^ responses at the systemic level.

### 3.4 *CNGC2* and *CNGC4* support spread of the wound-induced Ca^2+^ wave

To assess whether the altered systemic transcriptional response seen in the *CNGC2* and *CNGC4* mutants might reflect their role(s) in the propagation of the wound-triggered Ca^2+^ wave, we generated *cngc2–1* and *cngc4–1* lines expressing the GCaMP3 fluorescent Ca^2+^ biosensor and monitored the leaf-to-leaf and root-to-shoot systemic wound signaling. As noted in the methods, generating the reporter lines in these *CNGC* knockout backgrounds proved challenging, and so this analysis is limited to a single allele of both *CNGC2* and *CNGC4*.

In the leaf-to-leaf response, *cngc2–1* and *cng4–1* displayed a largely wild-type-like systemic Ca^2+^ wave, which initially spread through the vasculature and exhibited an increase in the systemic leaf approximately 20 s after wound stimulation and peaked at around 60 s (**Figures 3A, D, E**, **5**; **Supplementary Videos S7**, **S8**). Similarly, in both the *cngc2–1* and *cngc4–1* backgrounds, the root wound-induced Ca^2+^ wave resembled that of the wild type, moving along the root and being detectable in young and old leaves after approximately 20 s and peaking at 40–50 s, with rapid initial movement primarily through the vasculature (**Figures 4A, D, E**, **5**; **Supplementary Videos S9**, **S10**).

**Figure 6:**
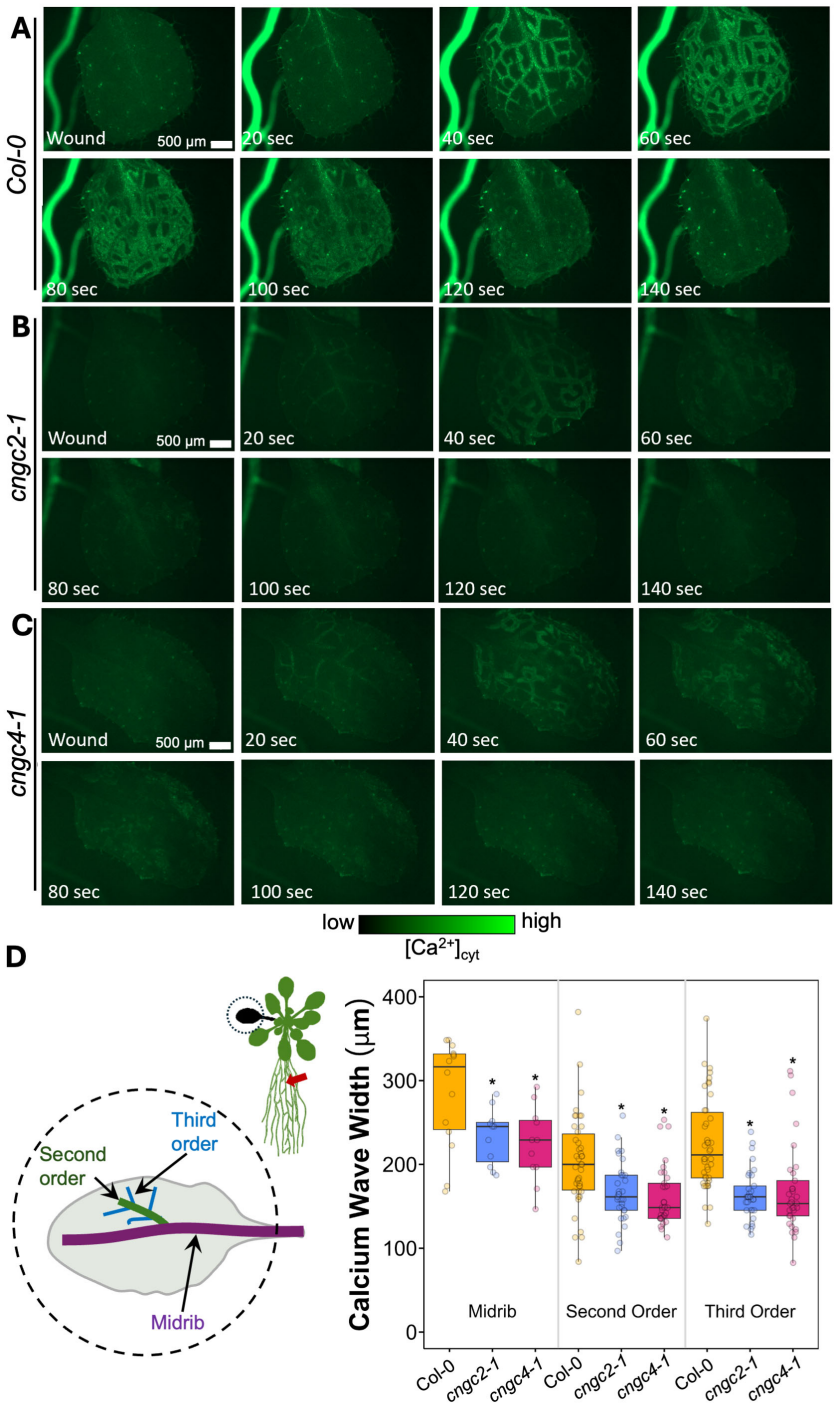
Ca^2+^ wave in systemic leaf in wild type, *cngc2-1*, and *cngc4-1*. After wounding of the root, a Ca^2+^ wave spreads through the leaves of Col-0 **(A)**, *cngc2-1* **(B)**, and *cngc4-1* **(C)** (see **Supplementary Videos S11** (Col-0), S12 (*cngc2-1*), and S13 (*cngc4-1*)). **(D)** Extent of the spread of the Ca^2+^ wave from the midrib vein, the second-order and third-order veins. Midrib, *n* = 11–14 separate leaves per genotype; second- and third-order *n* = 33–42 measurements from 11–14 separate leaves per genotype. The wavefront was determined as the furthest that the GCaMP fluorescence signal rose to >2 SD above the pre-stimulation levels (see **Supplementary Figure S9** for the measurement details). An asterisk indicates a significant difference in width (*p* < 0.05) from Col-0 at the same vein type based on Student’s *t*-test. Ca^2+^ levels were visualized by the GCaMP3 fluorescent biosensor.

However, we noticed that the Ca^2+^ response did appear to dissipate faster in the systemic leaves of the CNGC2 and CNGC4 mutants following root wounding than in the wild type (**Figures 5C, F, I**), with the caveat that such termination timings are heterogeneous even within a genotype. Along with the disruption of systemic induction of *JAZ5* and *JAZ7* in *cngc2–1* and *cngc4-1*, this observation led us to investigate the spread of the Ca^2+^ wave in more detail. Our observations that root wounding triggered a systemic Ca^2+^ response throughout the rosette allowed us to design an approach where we could focus at high magnification on a single leaf, apply a root wound stimulus, and then follow the dynamics of the ensuing systemic Ca^2+^ response in the target leaf. Critically, this methodology did not disturb the leaf’s position as the wound was applied, allowing us to record the essential pre-wound baseline data for the normalized F/F_0_ measurements that are needed to robustly monitor any wound-induced Ca^2+^ changes. When observed at these higher magnifications, differences became evident between the systemically responding leaves of Col-0, *cngc2-1*, and *cngc4-1*. As described above, when a wound-triggered Ca^2+^ increase arrives at a systemic leaf, the Ca^2+^ wave first travels through the vasculature. Upon arrival, the Ca^2+^ wave then spreads beyond the veins to fully traverse the tissue between the veins (**Figure 6A**). However, in *cngc2–1* and *cngc4-1*, we noted that although the Ca^2+^ wave traveled through the veins similarly to the wild type in both timing (**Figures 5A, D, G, J**) and intensity (**Supplementary Figure S10**), it appeared to only move a short distance beyond the vasculature relative to the wild-type response (**Figures 6B, C**; **Supplementary Videos S11–S13**). To quantify the extent of the spread of the Ca^2+^ wave from the vasculature, we measured the total width of the Ca^2+^ wave extending from either the main leaf vein (midrib or first-order leaf vein), the branches from the midrib (second-order leaf vein), or the secondary branches (third-order leaf vein). We set the baseline as the average fluorescence from that region of the systemic leaf before the wounding wave arrived. The extent of the spread of the Ca^2+^ wave was then defined as the furthest extent of the wavefront of wound-induced elevated GCaMP3 fluorescence that significantly rose above the 95% confidence limit (two standard deviations) of the variability in this baseline signal. The workflow for these measurements is outlined in **Supplementary Figure S9**. The total spread from the first-, second-, and third-order veins were all significantly reduced in *cngc2–1* and *cngc4-1* (*p* < 0.05; Student’s *t*-test), indicating that mutations in these genes restricted the spread of the wound-induced Ca^2+^ increases from the vasculature (**Figure 6D**). As the peak change in fluorescence of the midrib vein of the *cngc2–1* and *cngc4–1* leaves was not significantly different from the wild type (**Supplementary Figure S10**), the wound signal triggering the Ca^2+^ change appears to successfully travel through the vasculature, but defects in *cngc2–1* and *cngc4–1* then prevent the subsequent Ca^2+^ change from fully spreading after arrival.

The resting fluorescence from the *cngc2–1* and *cngc4–1* reporter lines was lower than that of the wild type (**Supplementary Figure S11**), as was the absolute magnitude of the wound-induced increase (**Figures 3A**, **4A**, **6A–C**; **Supplementary Figure S8**). This reduced signal was not due to differences in reporter expression levels between lines (**Supplementary Figure S12**) and is consistent with the observation that CNGC2 likely forms a Ca^2+^ channel mediating basal Ca^2+^ influx in these plants (Wang et al., 2017). Therefore, Ca^2+^ levels and influx associated with wound-induced Ca^2+^ wave propagation may also be reduced when this channel is not functional. However, as a note of caution, comparing absolute quantitative differences in responses between Ca^2+^ reporter lines can be complex even when the results are normalized to initial fluorescence levels (i.e., the F/F_0_ reported herein). Thus, while differences in kinetics of response and spatial patterning are likely robust, inferences as to apparent reductions in the magnitude of response should be viewed as tentative.

## 4 Discussion

### 4.1 Mutants in many CNGCs do not significantly disrupt systemic wound responses

Of the 17 members of the CNGC family tested, we found only two—CNGC2 and CNGC4—altered systemic signaling both at the level of the spread of the wound-induced Ca^2+^ wave (**Figures 6A–D**) and systemic transcriptional responses (**Figures 1** and **2**). CNGC19 was initially a promising candidate for playing a role in this system as it is known to be important for mediating defense responses to herbivory and to support the local spread of the Ca^2^ *^+^* wave at the site of herbivore damage (Meena et al., 2019). However, both the systemic spread of the wound-triggered Ca^2^ *^+^* wave beyond the damaged leaf or root (**Figures 3A–C**, **4A–C**) and the transcriptional responses elicited in leaf-to-leaf and root-to-shoot systemic signaling (**Figures 1A–C**, **2A–C**; **Supplementary Figures S3**, **S4**) were similar to the wild type in *CNGC19* knockout lines. These observations suggest that CNGC19 is not essential for the systemic wound responses that we have monitored, so either there are likely differences between the molecular machinery supporting local and the systemic wound response or CNGC19 may exert rapid effects not resolved with the 40-min timepoint we have used to monitor molecular changes. In addition, we optimized our leaf wounding protocol to impose a crush wound with serrated tweezers rather than the cutting damage imposed by Meena et al. (2019). We reasoned that as the crush wound covers a larger area of the leaf than a cut, it is easier to reproducibly apply. This approach then aided in minimizing differences in the elicitation of the wound response between experiments, helping make comparisons between genotypes more robust. However, the much more extensive wound signal triggered by this stimulus likely masks any subtler *CNGC19* effects in the directly damaged leaves with *cngc19-1* (and *cngc20*) attaining a wild-type-like peak in fluorescence intensity in the crush-damaged leaf (**Supplementary Figure S8A**).

CNGC20, the closest channel family member to CNGC19 within the CNGC Group Iva, is thought to form heteromeric channels with CNGC19 (Yuen and Christopher, 2013; Yu et al., 2019). However, *cngc20* also behaved similarly to the wild type in terms of both root-to-shoot and leaf-to-leaf systemic Ca^2+^ wave propagation (**Figures 3A–C**, **4A–C**) and systemic transcriptional responses (**Figures 1A–C**, **2A–C**; **Supplementary Figures S3**, **S4**). Furthermore, mutants in *CNGC20* behave as the wild type in insect herbivory trials (Meena et al., 2019). Thus, CNGC20 is more likely playing roles specific to other aspects of growth, development, and/or signaling than supporting the systemic spread of the wound response, with, e.g., this channel recently being shown to play a key role in freezing tolerance (Peng et al., 2024).

CNGC17 was another strong candidate for mediating systemic signaling, as it is known to be part of a complex which includes multiple defense-related proteins and the *Arabidopsis* H^+^-ATPase AHA1 (Ladwig et al., 2015). AHA1 plays an important role in the vascular propagation of long-distance electrical signals during systemic wound signaling (Kumari et al., 2019). However, as with the *CNGC19* knockouts, we found that the *cngc17* mutant had wild-type-like induction of systemic leaf-to-leaf and root-to-shoot transcriptional responses.

As a note of caution, our screen for the roles of CNGC family members in systemic signaling, with the exception of *cngc10/13*, has relied upon phenotyping mutants of individual genes. Therefore, the roles of some of the CNGCs may have been masked by functional redundancy between family members. Although an analysis of multiple knockouts in closely related CNGCs would be a powerful approach to address this possibility, our screening of transcriptional response shown in **Figures 1**, **2** and **Supplementary Figures S3–S6** does not provide clear hints as to the additional candidate groups to target, making these higher-order mutants beyond our focus on Group IVb (CNGC2 and CNGC4).

### 4.2 *CNGC2* and *CNGC4* act in the spread of the Ca^2+^ wave in systemic tissues

The Ca^2+^ channels currently most closely linked with systemic transmission of the wound response are the GLRs, with *GLR3.3* and *GLR3.6* being critical for the spread of systemic wound response (reviewed in Johns et al. (2021)). These channels are expressed in the phloem and xylem contact cells, respectively (Nguyen et al., 2018; Moe-Lange et al., 2021; Wu et al., 2024), implying that the action of a network of other channels and transporters must then be acting to propagate Ca^2+^ responses throughout the rest of the plant’s tissues. Indeed even at the level of the vasculature, the mechanosensitive anion channel MSL10 has been shown to act alongside the GLRs in propagating the systemic Ca^2+^ signal (Moe-Lange et al., 2021). Our analyses suggest that CNGC2 and CNGC4 may be a part of this system, likely acting to spread the systemic response beyond the vascular tissues.

Thus, following leaf and root wounding, the systemic induction of the wounding response markers *JAZ5* and *JAZ7* in both leaf-to-leaf or root-to-rosette systemic wounding response was reduced by more than 50% in *cngc2-1*, *cngc2-2*, *cngc4-1*, and *cngc4-3* (**Figures 1** and **2**). Superficially, the Ca^2+^ wave in *cngc2–1* and *cngc4–1* displayed a similar patterning to the wild type, propagating rapidly from the wound site through the vasculature to the systemic leaves (**Figures 3A, D, E**, **4A, D, E**, **5**). However, when focusing more closely on the kinetics in individual systemic leaves, the spread of the Ca^2+^ wave through the lamina from the midrib and second- and third-order vascular branches is significantly reduced in *cngc2–1* and *cngc4-1* (**Figure 6D**). *CNGC2* and *CNGC4* are ubiquitously expressed throughout the plant (Berrío et al., 2022), including across the leaf lamina. Thus, these channels would be ideally placed to act to spread the response to the arrival of systemic signaling molecules such as β-thioglucoside glucohydrolases, glutamate, and glutathione that are drawn through the vasculature from the wound site to arrive in the veins of the systemic leaves (Bellandi et al., 2022; Gao et al., 2023, 2024) or to hydraulic signals propagating through vascular tissues (Grenzi et al., 2023).

Indeed CNGC2 has been previously identified as a Ca^2+^ influx channel responsible for loading Ca^2+^ into the mesophyll cells from the apoplast (Wang et al., 2017, 2024), fitting well with a role supporting the spread of systemic signals across the leaf. CNGC2 has also been implicated in the plant-wide signaling to local high light stress through a network including reactive oxygen species-dependent signaling (Fichman et al., 2021). Here CNGC2 was proposed to act to amplify responses to a propagating wave of ROS, implying a potentially broader role for this channel in systemic response networks that spread multiple signals throughout the plant body. Indeed such reactive oxygen species-driven systemic signaling from wounding and heat stress (although not high light stress) is known to have both vascular and non-vascular propagation pathways (Zandalinas and Mittler, 2021). Furthermore, CNGC2 and CNGC4 are known to play central roles in plant immune signaling (Yu et al., 1998; Jurkowski et al., 2004) and recently have been shown to act in the plant’s thermotolerance response system (Ma et al., 2023), potentially through mechanisms independent of their roles in defense (Lu et al., 2022). Although CNGC2 and CNGC4 physically interact with each other to form a multimeric channel (Tian et al., 2019), they also interact with CNGC6, and all three play roles in defense signaling (Ma et al., 2023). However, we found that *cngc6* mutants do not affect the systemic responses where CNGC2 and CNGC4 play a key role. These observations all raise the key question of how far these channels are generally supporting Ca^2+^ influx across tissues versus acting in signaling networks that promote the systemic spread of specific responses tuned to the action of specific CNGCs.

## Data Availability

The datasets presented in this study can be found in online repositories. The names of the repository/repositories and accession number(s) can be found in the article/**Supplementary Material**.
