# Supplementary material for: The cyclic nucleotide-gated channels CNGC2 and CNGC4 support systemic wound responses in Arabidopsis thaliana
Source: Front Plant Sci. 2025 Aug 22;16:1545065. doi: 10.3389/fpls.2025.1545065 (PMC12411437; doi:10.3389/fpls.2025.1545065)
Supplement: Supplementary file 14 [file DataSheet1.pdf]

Supplementary Material

1. Supplementary Figures

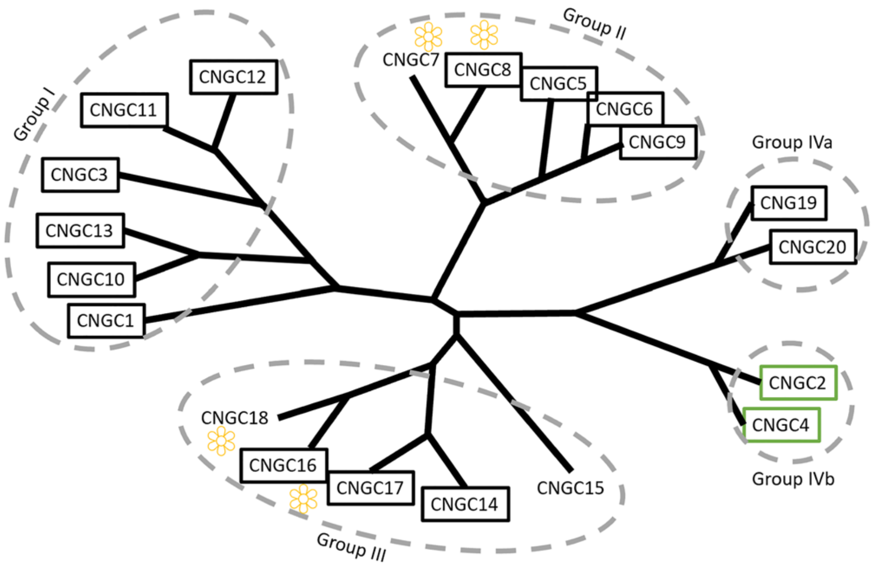

Supplementary Figure 1. CNGC family. As adapted from Maser et al. (2001), the CNGCs are divided into 5 groups based on sequence similarity. Black outline indicates knockout mutants had a response similar to wild type in all experiments conducted. Green outline indicates a significant difference from wild type. Yellow flower indicates expression only in pollen. Mutants in CNGC7, CNGC15, and CNGC18 were not tested in this study.

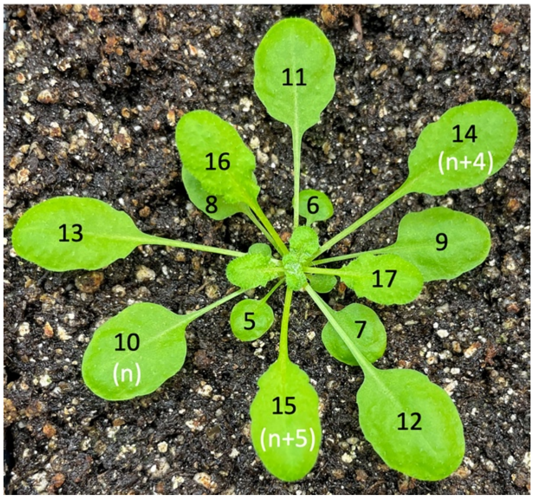

Supplementary Figure 2. Leaf organization in *Arabidopsis thaliana*. Leaves are numbered (black) in order of development, with the highest numbers being the youngest leaves. For systemic wounding assays, leaf (n) is wounded and leaves (n+4) and (n+5) are collected (white).

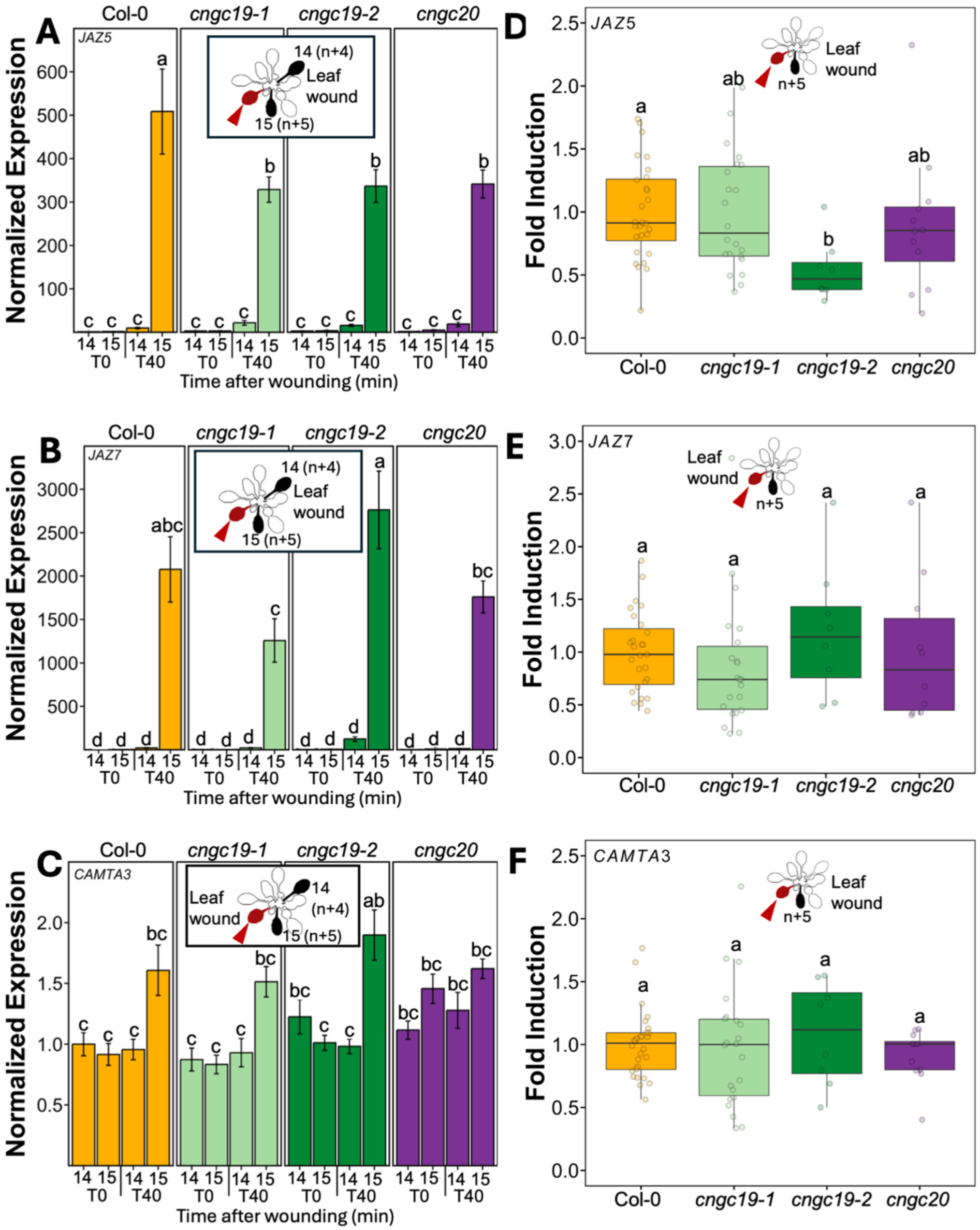

Supplementary Figure 3: Mutants in *CNGC19* and *CNGC20* do not disrupt systemic transcriptional responses to leaf wounding. (A-C) Following wounding of leaf 10, the expression in leaves 14 and 15 of *JAZ5* (A), *JAZ7* (B), and *CAMTA3* (C) as wounding response markers was measured in *cngc19-1, cngc19-2,* and *cngc20* at 0 (T0) and 40 (T40) min post wounding. Expression is normalized to Col-0 leaf 14 at 0 min post wounding. Data is mean ± SEM of 3-4 biological replicates with 3 technical replicates of a representative experiment. (D-F) Expression of *JAZ5* (D), *JAZ7* (E), and *CAMTA3* (F) in leaf 15 at 40 min across 2-4 independent experiments (8-19 separate plants) normalized as fold induction relative to the Col-0 level. Bars or plots with different letters indicate significant difference ($p<0.05$) based on ANOVA with multiple comparisons via Tukey. Note, (D)-(F) use the same data as in Figure 1 to aid with comparisons.

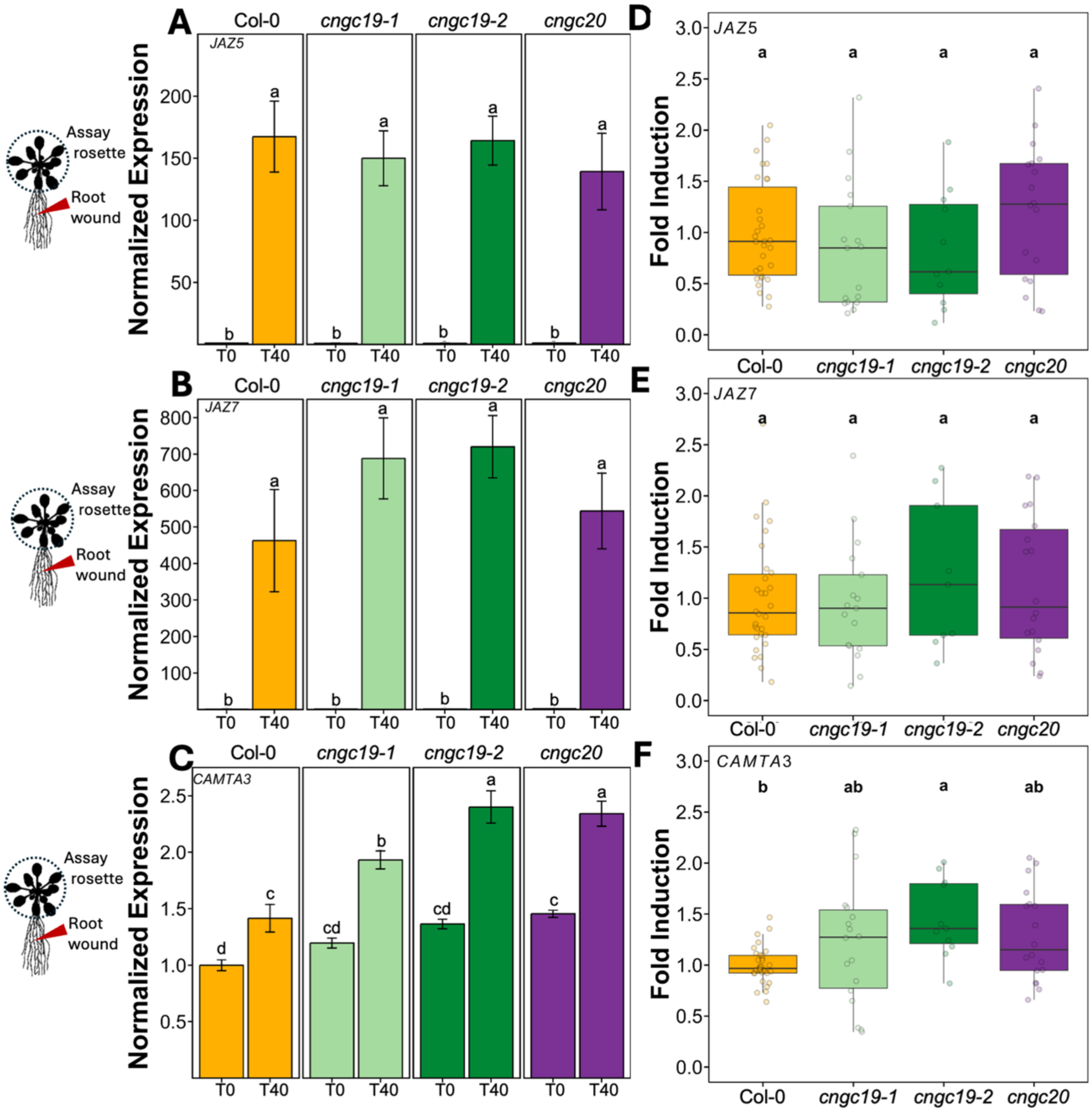

Supplementary Figure 4: Mutants in *CNGC19* and *CNGC20* do not disrupt systemic transcriptional responses to root wounding. (A-C) Following wounding of the root, the expression in the shoot of *JAZ5* (A), *JAZ7* (B), and *CAMTA3* (C) as wounding response markers was measured in *cngc19-1, cngc19-2,* and *cngc20* at 0 (T0) and 40 (T40) min post wounding. Expression is normalized to Col-0 at 0 min post wounding. Data is mean ± SEM of 3-4 biological replicates with 3 technical replicates of a representative experimental replicate. (D-F) Expression of *JAZ5* (D), *JAZ7* (E), and *CAMTA3* (F) at 40 min was normalized as fold induction relative to the Col-0 level for 3-4 experimental replicates (n=10-12). Bars or plots with different letters indicate significant difference ($p<0.05$) based on ANOVA with multiple comparisons via Tukey. Note, (D)-(F) use the same data as in Figure 2 to aid with comparisons.

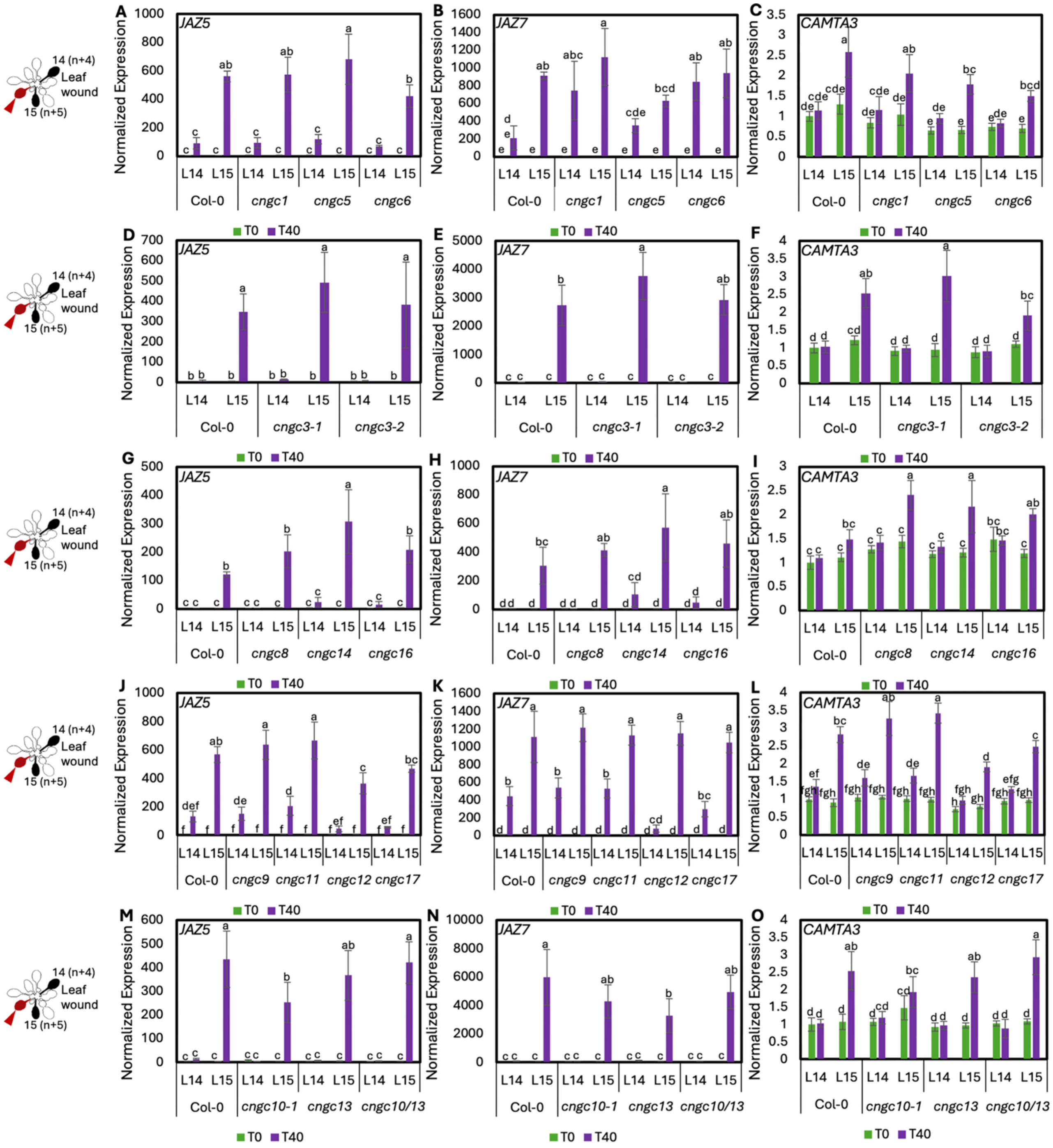

Supplementary Figure 5: Most CNGCs are not required for systemic leaf signaling. . Systemic expression of *JAZ5*, *JAZ7*, and *CAMTA3* following wounding of leaf 10 monitored at 0 (T0) and 40 (T40) min post wounding in *cngc1* (A-C), *cngc3-1* (D-F), *cngc3-2* (D-F), *cngc5* (A-C), *cngc6* (A-C), *cngc8* (G-I), *cngc9* (J-L), *cngc10-1* (M-O), *cngc10/13* (M-O), *cngc11* (J-L), *cngc12* (J-L), *cngc13* (M-O), *cngc14* (G-I), *cngc16* (G-I), and *cngc17* (J-L). Expression is normalized to Col-0 leaf 14 at 0 min post wounding. Data is mean ± SEM of 3-4 biological replicates with 3 technical replicates of a representative experimental replicate from Figure 1. Plots with different letters indicate significant difference ($p<0.05$) based on ANOVA with multiple comparisons via Tukey.

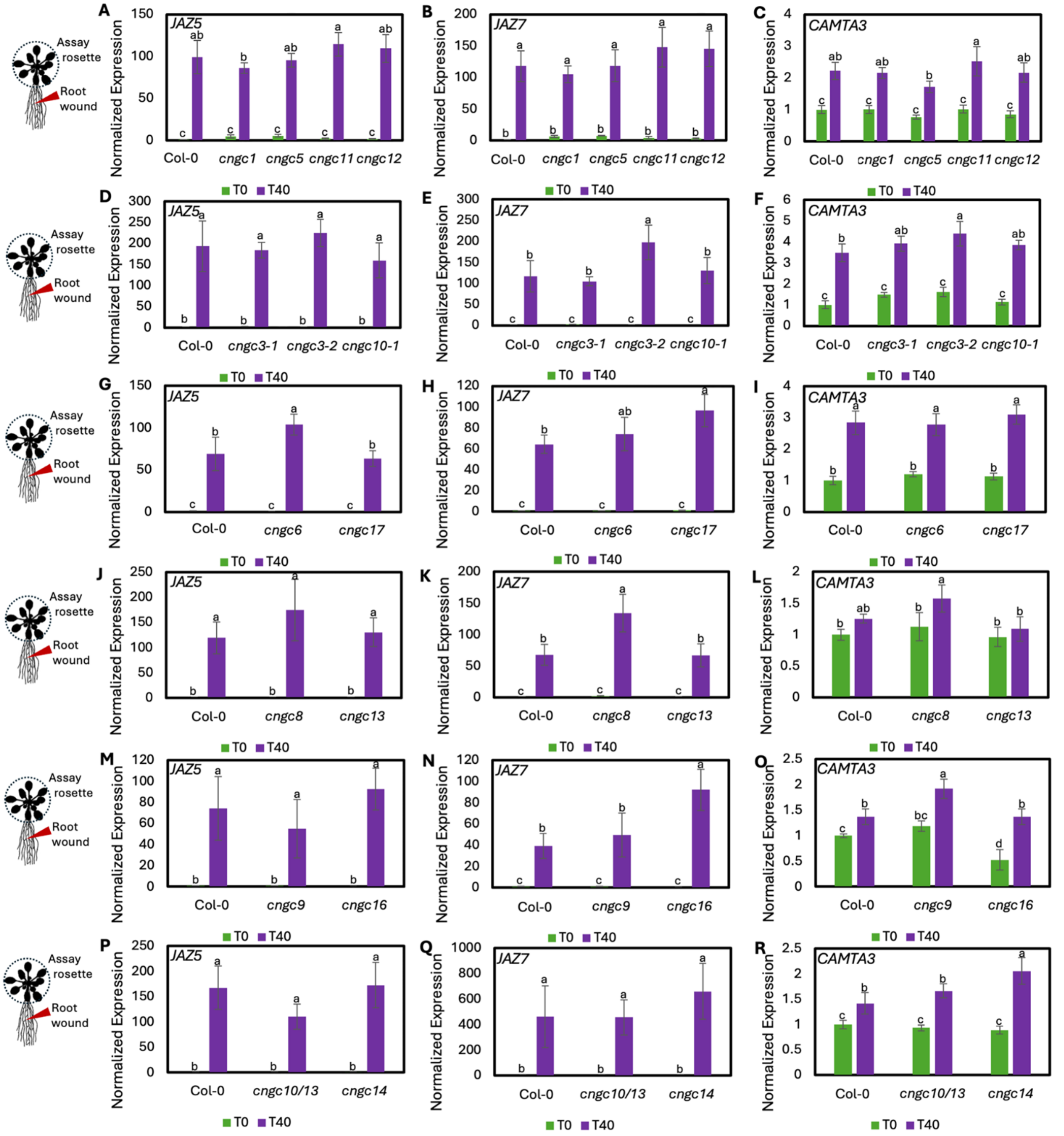

Supplementary Figure 6: Most CNGCs are not required for root-to-shoot signaling. Following wounding of the root, the expression in the shoot of *JAZ5*, *JAZ7*, and *CAMTA3* as wounding response markers was measured at 0 (T0) and 40 (T40) min post wounding in *cngc1* (A-C), *cngc3-1* (D-F), *cngc3-2* (D-F), *cngc5* (A-C), *cngc6* (G-I), *cngc8* (J-L), *cngc9* (M-O), *cngc10-1* (D-F), *cngc10/13* (P-R), *cngc11* (A-C), *cngc12* (A-C), *cngc13* (J-L), *cngc14* (P-R), *cngc16* (M-O), and *cngc17* (G-I). Expression is normalized to Col-0 at 0 min post wounding. Data is mean ± SEM of 3-4 biological replicates with 3 technical replicates of a representative experimental replicate from Figure 2. Plots with different letters indicate significant difference ($p<0.05$) based on ANOVA with multiple comparisons via Tukey.

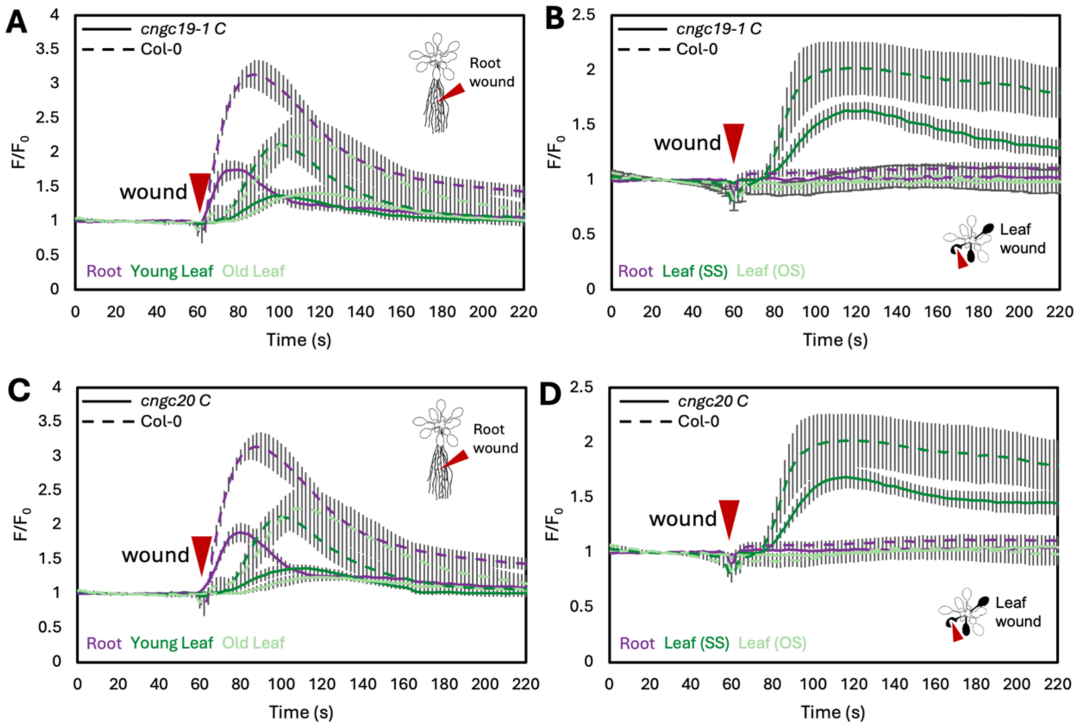

Supplementary Figure 7: Ca^{2+} wave following wounding in *cngc19-1* and *cngc20* in a root or leaf. Experiment identical to that presented in Figures 3 and 4 but using an independent GCaMP3 Ca^{2+} reporter line produced by crossing to the GCaMP3 expressing wild-type line rather than an independent transformation for *cngc19-1* and *cngc20* as shown in figures 3 and 4. Quantification of normalized fluorescence (F/F_0) after wounding the main root in *cngc19-1* (A) and *cngc20* (C). Data are mean ± SE, n=8-13. See Supplementary Videos 14 and 16. An increase in F/F_0 represents and increase in Ca^{2+} level.. Quantification of normalized fluorescence (F/F_0) after leaf wounding *cngc19-1* (B) and *cngc20* (D) (mean ± SE, n=8-11). See Supplementary videos 15 and 17. Solid lines indicate mutants and dashed lines represent the same Col-0 control data in each panel for comparison (this control data is also shown in Figures 3 and 4). F = fluorescence intensity at timepoint; F_0 = average fluorescence before wound stimulus.

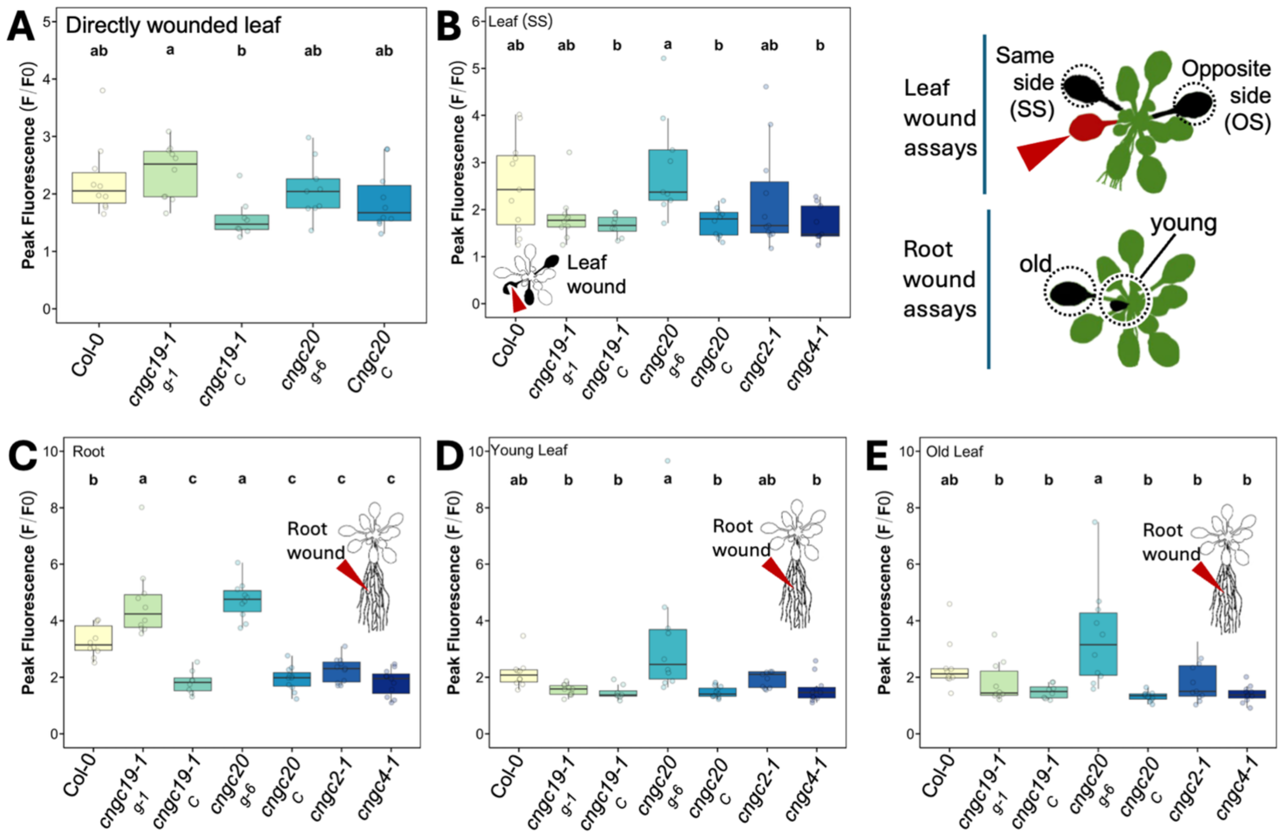

Supplementary Figure 8: Peak Fluorescence of the Ca^{2+} wave following leaf or root wounding. Following leaf wounding, the peak fluorescence value (F/F_0) was measured in the (A) wounded leaf and (B) Leaf (SS) for *cngc19-1 g-1*, *cngc19 C*, *cngc20 g-6*, *cngc20 C*, *cngc2-1*, and *cngc4-1* (lines labeled g are transformed, lines labeled C were crossed with the wild-type Col-0 expressing GCaMP3). Data corresponds to panels and graphs from Figure 3 and Supplementary Figure 7. Following root wounding, the peak fluorescence value was measured in the (C) root, (D) young leaf, and (E) old leaf for *cngc19-1 g-1*, *cngc19 C*, *cngc20 g-6*, *cngc20 C*, *cngc2-1*, and *cngc4-1*. Data corresponds to panels and graphs from Figure 4 and Supplementary Figure 5. F = fluorescence intensity at timepoint; F_0 = average fluorescence before wound stimulus.

1. Opened timelapse files (from AxioZoom microscope in Zeiss .czi format) in ImageJ and ran the StackReg plugin with Rigid Body transformation to best align the individual images.
2. Measured a rectangle of background mean gray value across the vein to be analyzed and calculated the average and standard deviation of background pre-wounding.
3. Drew a line across the vein perpendicular to the vein's main axis and used the Plot Profile analysis tool to measure pixel intensities across the transect.
4. Used the average of background + 2 StdDev as a cutoff to find the edges of the peak at X_1 and X_2. Calculated X_2-X_1 for total calcium wave width at that timepoint. Repeated every measurement every 10 s until the pixel values fell back to below the background +2 StdDev value. Used the largest X_2-X_1 value for statistical analysis.

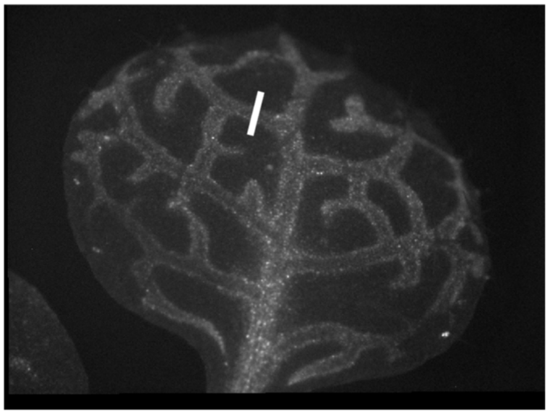

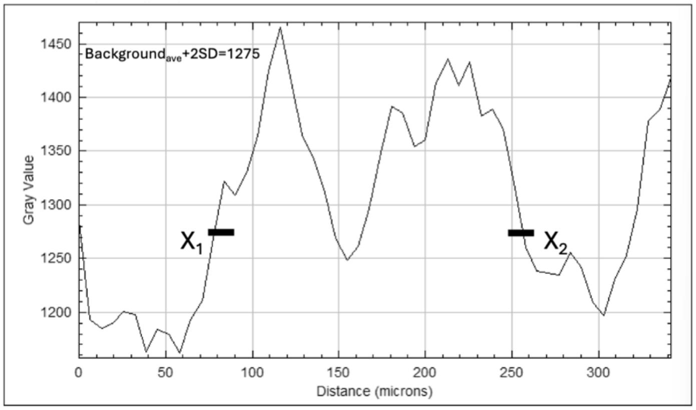

Supplementary Figure 9: Workflow to determine maximum calcium wave width.

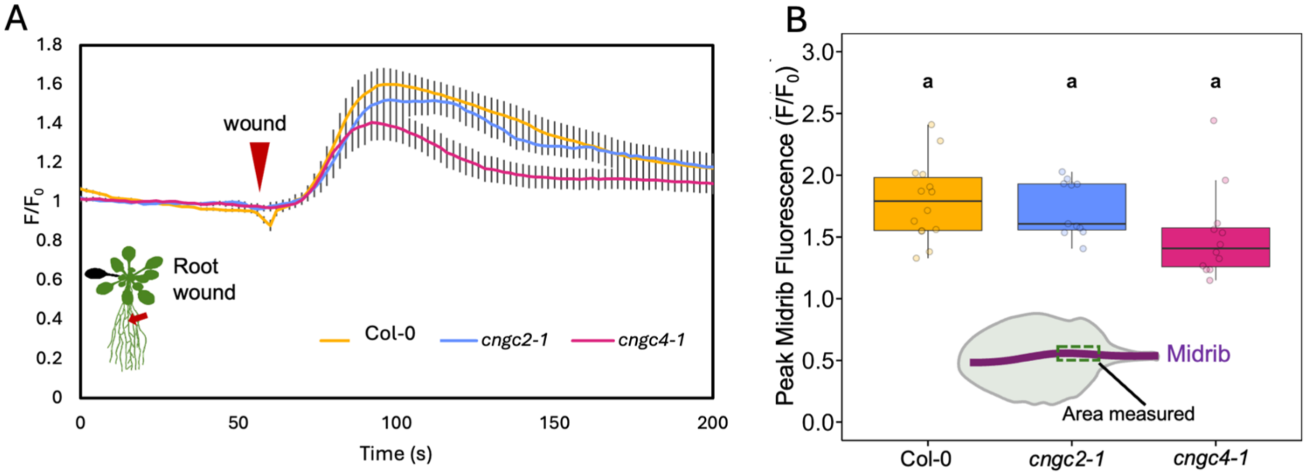

Supplementary Figure 10: Peak fluorescence of midrib veins. (A) Normalized fluorescence (F/F_0) of Ca^{2+} changes in the midrib vein after wounding the main root in Col-0, *cngc2-1,* and *cngc4-1* as seen in Figure 6. (B) Quantification of the peak fluorescence values in the midrib vein. Data corresponds to panels and graphs from Figure 6. F = fluorescence intensity at timepoint; F_0 = average fluorescence before wound stimulus.

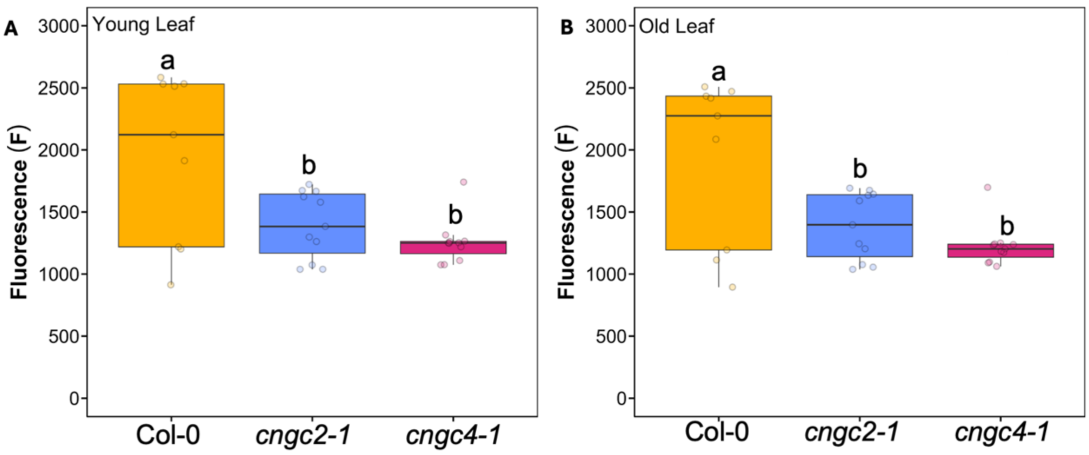

Supplementary Figure 11: Absolute fluorescence intensity before wounding in Col-0, *cngc2-1* and *cngc4-1*. Fluorescence (F) of pre-wounded young and old leaves in Col-0, *cngc2-1*, and *cngc4-1* averaged across the whole leaf over 40 sec. Data corresponds to Figure 4 pre-root wound mean gray scale values in young (A) and old (B) leaves. Bars sharing the same letter are not significantly different (*p<0.05*) based on ANOVA with multiple comparisons via Tukey.

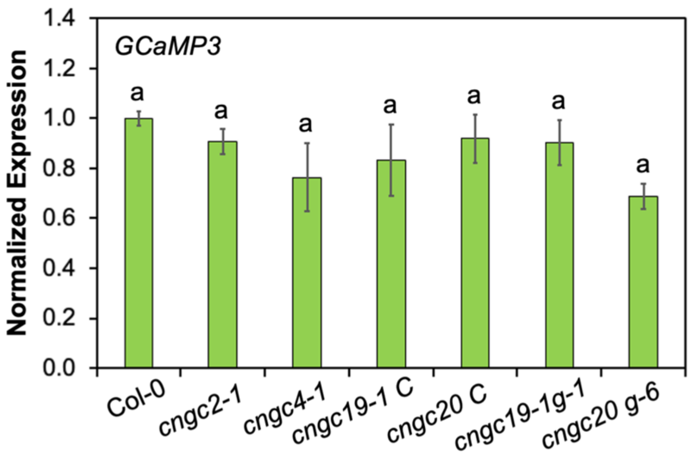

Supplementary Figure 12: GCaMP3 reporter expression in wild type, *cngc2*, *cngc4*, *cngc19*, and *cngc20* mutant lines. GCaMP3 expression was analyzed by qPCR normalized using *UBQ10*. Results are mean ± s.d., n=6 per genotype. Bars sharing the same letter are not significantly different according to one-way ANOVA and Tukey Kramer, $p<0.05$. Ca^{2+} measurements made using the GCaMP3 fluorescent biosensor. *cngc19-1 g-1 and cngc20 g-6* were lines independently transformed with the reporter. All other lines were generated by crossing to the Col-0 GCaMP3 reporter line.

2. Supplementary Tables

Supplementary Table 1: Mutant lines used in this study.

Mutant	Line	Reference
cngc1	SAIL_443_B11 (CS874223)	(Yu et al., 2019)
cngc2-1	SALK_019922C	(Katano et al., 2018)
cngc2-2	SALK_066908C	(Katano et al., 2018)
cngc3-1	SALK_056832	(Gobert et al., 2006; Yu et al., 2019)
cngc3-2	SALK_066634	(Gobert et al., 2006)
cngc4-1	SALK_081369	(Chin et al., 2013)
cngc4-3	CS6524	(Jurkowski et al., 2004)
cngc5	SALK_149893C	(Yu et al., 2019)
cngc6-1	SALK_042207	(Brost et al., 2019; Yu et al., 2019)
cngc8	SALK_201666C	Pollen expressed
cngc9	SALK_026086	(Yu et al., 2019)
cngc10-1	WiscDsLoxHs080_05B	Confirmed T-DNA insertion in exon.
cngc11	SALK_026568C	(Yu et al., 2019)
cngc12	SALKseq_4705.1	Confirmed T-DNA insertion in exon.
cngc13	SALK_057742C	(Moon et al., 2019)
cngc14-4	SALKSEQ_086744	(Brost et al., 2019)
cngc16	SALK_065792	Pollen expressed (Moon et al., 2019)
cngc17	SALK_111237C	Confirmed T-DNA insertion in exon.
cngc19-1	SALK_027306	(Meena et al., 2019)
cngc19-2	SALK_129200C	(Meena et al., 2019)
cngc20-1	SALK_129133	(Yu et al., 2019)
cngc10/13	*cngc10-2* SAILseq_853_C05<br><br>*cngc13-2* SALK_013536<br>Gift from Keiko Yoshioka (University of Toronto)	Confirmed by qPCR by the Yoshioka Lab (unpublished)<br>(Moon et al., 2019)

Supplementary Table 2: Genotyping primers used in this study.

Gene	Mutant	Direction	Primer
CNGC1	SAIL_443_B11	LP	CAAGCTCTGCAAGGATCAAAC
CNGC1	SAIL_443_B11	RP	TAGAAATGAACACCGCGAAAC
CNGC2-1	SALK_019922C	LP	TTCGGCACAACTAACCAAAAC
CNGC2-1	SALK_019922C	RP	CTTTTCCCGGAAAATTCACTC
CNGC2-2	SALK_066908C	LP	ATATCCAACTGTGCTTGTCGG
CNGC2-2	SALK_066908C	RP	CTTTCCATTCAACTAGCTGCG
CNGC3-1	SALK_056832	LP	AAATCAGAACCTTTAAGCGGC
CNGC3-1	SALK_056832	RP	TACCAAAGTTGAAAACCGTCG
CNGC3-2	SALK_066634	LP	CTGTTGTGGCTTTAGCCTTTG
CNGC3-2	SALK_066634	RP	CACTCGTCTTCAAGTTTTGGC
CNGC4-1	SALK_081369	LP	CTGTTGTGCTCTCCAAATTCC
CNGC4-1	SALK_081369	RP	TCACATGGACCTTTTCCATTG
CNGC5	SALK_149893C	LP	GAGCTTTCTGGTTAAGCCGTC
CNGC5	SALK_149893C	RP	CACGCTCCCTAAGATCTTGTG
CNGC6-1	SALK_042207	LP	TCCAGGATATGTGCTGGTTTC
CNGC6-1	SALK_042207	RP	TCCGTTGATCCTCTCTTCTTG
CNGC8	SALK_201666C	LP	TCAGGGACTGATTTGATCCAG
CNGC8	SALK_201666C	RP	AAGCGATCATGTTTTGGATTG
CNGC9	SALK_026086	LP	ATTTGCAGCAAACTTTGAAGC
CNGC9	SALK_026086	RP	TGTTTATGGTGGGGACTTCAG
CNGC10-1	WiscDsLoxHs080_05B	LP	ATCCACTGCTCTGCATCTCTC
CNGC10-1	WiscDsLoxHs080_05B	RP	CTATCCTTCCTCTCCCACAGG
CNGC11	SALK_026568C	LP	TTTACAGTGGTTGAGGTGGTG
CNGC11	SALK_026568C	RP	TTTTGTTTTGTTTCAGGTGCC
CGNC12	SALKseq_4705.1	LP	CGGTTGTACTTTTCAGCTTGC
CNGC12	SALKseq_4705.1	RP	CACCCACTGTTGTTGTTGTTG
CNGC13	SALK_057742C	LP	TTTTGGTCAAAACACACACATG
CNGC13	SALK_057742C	RP	CAGCTATGCAATTCGAGAAGG
CNGC14-4	SALKSEQ_086744	LP	AAACTGCTGAACATTTTTGTGG
CNGC14-4	SALKSEQ_086744	RP	GCCATCTCTGTCTCGATCTTG
CNGC16	SALK_065792	LP	TTTGGTTTGCACAAAATCATG
CNGC16	SALK_065792	RP	TTTAACCTGGGCTTTAGTGCC
CNGC17	SALK_111237C	LP	TGGGACAAAGAAGTACAACGG
CNGC17	SALK_111237C	RP	TCGTTTTCTGATTGGAATGTG
CNGC19-1	SALK_027306	LP	CGCGGATCTCTTTATTCACAC
CNGC19-1	SALK_027306	RP	ATGAGGATTCATTATTCCGGG
CNGC19-2	SALK_129200C	LP	AGGAGGGTGAGAGAGGTTGAG
CNGC19-2	SALK_129200C	RP	CCAAGTTACTCAAGCCGAGTG
CNGC20-1	SALK_129133	LP	AAAACAGTTACCTGGAAGCCC
CNGC20-1	SALK_129133	RP	TGCCTTTACACCACCTTTTTG

SALK (LBb1.3)			ATTTTGCCGATTTCGGAAC
SAIL			TAGCATCTGAATTTCATAACCAATCTCGATACAC
WiscDsLoxHs			TGATCCATGTAGATTTCCCGGACATGAAG

Supplementary Table 3: RT-qPCR primers used in this study. FP, forward primer; RP, reverse primer.

Gene	Direction	Primer
UBQ10	FP	CACACTCCACTTGGTCTTGCGT
UBQ10	RP	TGGTCTTTCCGGTGAGAGTCTTCA
JAZ5	LP	TCATCGTTATCCTCCCAAGC
JAZ5	RP	CACCGTCTGATTTGATATGGG
JAZ7	LP	GATCCTCCAACAATCCCAAA
JAZ7	RP	TGGTAAGGGGAAGTTGCTTG
CAMTA3	FP	TTCCGAGGTTACAAGGGAAG
CAMTA3	RP	CCCCCACTGACCAAATTATC
GCaMP3	FP	CTCACTCGAGAACGTCTATATCAAGGC
GCaMP3	RP	CGATGTTGTGGCGGATCTTGAAGTTC

3. Supplementary Videos

Supplementary Video 1. Ca^{2+} wave following leaf wounding. One leaf was wounded using tweezers in wild type *Arabidopsis thaliana*, expressing the calcium sensor GCaMP3. GCaMP3 exhibits increased fluorescence with an increase in calcium. Images were taken every 2 s and the movie represents 14X speed.

Supplementary Video 2. Ca^{2+} wave following root wounding. The main root was wounded using tweezers in wild type *Arabidopsis thaliana*, expressing the calcium sensor GCaMP3, which exhibits increased fluorescence with an increase in calcium. Images were taken every 2 s and the movie represents 14X speed.

Supplementary Video 3. Ca^{2+} wave following leaf wounding in *cngc19-1*. One leaf was wounded using tweezers in *cngc19-1*. expressing the calcium sensor GCaMP3. GCaMP3 exhibits increased fluorescence with an increase in calcium. Images were taken every 2 s and the movie represents 14X speed. Brightness was linearly increased in the video using ImageJ post any quantitative analysis for ease of viewing.

Supplementary Video 4. Ca^{2+} wave following root wounding in *cngc19-1*. The main root was wounded using tweezers in *cngc19-1* expressing the calcium sensor GCaMP3. GCaMP3 exhibits increased fluorescence with an increase in calcium. Images were taken every 2 s and the movie represents 14X speed. Brightness was linearly increased in the video using ImageJ post any quantitative analysis for ease of viewing.

Supplementary Video 5. Ca^{2+} wave following leaf wounding in *cngc20-1*. One leaf was wounded using tweezers in *cngc20-1*expressing the calcium sensor GCaMP3. GCaMP3 exhibits increased fluorescence with an increase in calcium. Images were taken every 2 s and the movie represents 14X speed.

Supplementary Video 6. Ca^{2+} wave following root wounding in *cngc20-1.* The main root was wounded using tweezers in *cngc20-1* expressing the calcium sensor GCaMP3. GCaMP3 exhibits increased fluorescence with an increase in calcium. Images were taken every 2 s and the movie represents 14X speed.

Supplementary Video 7. Ca^{2+} wave following leaf wounding in *cngc2-1*. One leaf was wounded using tweezers in *cngc2-1* expressing the calcium sensor GCaMP3. GCaMP3 exhibits increased fluorescence with an increase in calcium. Images were taken every 2 s and the movie represents 14X speed.

Supplementary Video 8. Ca^{2+} wave following leaf wounding in *cngc4-1*. One leaf was wounded using tweezers in *cngc4-1* expressing the calcium sensor GCaMP3. GCaMP3 exhibits increased fluorescence with an increase in calcium. Images were taken every 2 s and the movie represents 14X speed. Brightness was linearly increased in the video using ImageJ post any quantitative analysis for ease of viewing.

Supplementary Video 9. Ca^{2+} wave following root wounding in *cngc2-1*. The main root was wounded using tweezers in *cngc2-1* expressing the calcium sensor GCaMP3. GCaMP3 exhibits increased fluorescence with an increase in calcium. Images were taken every 2 s and the movie represents 14X speed. Brightness was linearly increased in the video using ImageJ post any quantitative analysis for ease of viewing.

Supplementary Video 10. Ca^{2+} wave following root wounding in *cngc4-1*. The main root was wounded using tweezers in *cngc4-1* expressing the calcium sensor GCaMP3. GCaMP3 exhibits increased fluorescence with an increase in calcium. Images were taken every 2 s and the movie represents 14X speed.

Supplementary Video 11. Ca^{2+} wave in one leaf following root wounding. The main root was wounded using tweezers in wild type *Arabidopsis thaliana* expressing the calcium sensor GCaMP3. GCaMP3 exhibits increased fluorescence with an increase in calcium. Images were taken every 2 s and the movie represents 14X speed. Brightness was linearly increased in the video using ImageJ post any quantitative analysis for ease of viewing.

Supplementary Video 12. Ca^{2+} wave in one leaf following root wounding in *cngc2-1*. The main root was wounded using tweezers in *cngc2-1* expressing the calcium sensor GCaMP3. GCaMP3 exhibits increased fluorescence with an increase in calcium. Images were taken every 2 s and the movie represents 14X speed. Brightness was linearly increased in the video using ImageJ post any quantitative analysis for ease of viewing.

Supplementary Video 13. Ca^{2+} wave in one leaf following root wounding in *cngc4-1*. The main root was wounded using tweezers in *cngc4-1* expressing the calcium sensor GCaMP3. GCaMP3 exhibits increased fluorescence with an increase in calcium. Images were taken every 2 s and the movie represents 14X speed. Brightness was linearly increased in the video using ImageJ post any quantitative analysis for ease of viewing.
